# Sniffer restricts arboviral brain infections by regulating ROS levels and protecting blood-brain barrier integrity in *Drosophila* and mosquitoes

**DOI:** 10.1371/journal.ppat.1012797

**Published:** 2024-12-16

**Authors:** Rui Hu, Mengzhu Li, Shulin Chen, Man Wang, Xinjun Tao, Yihan Zhu, Huan Yan, Yuan Liu

**Affiliations:** State Key Laboratory of Virology, College of Life Sciences, Wuhan University, Wuhan, China; Institut Pasteur, FRANCE

## Abstract

Arthropod-borne viruses (arboviruses) are transmitted to humans by arthropod vectors and pose a serious threat to global public health. Neurotropic arboviruses including Sindbis virus (SINV) persistently infect the central nervous system (CNS) of vector insects without causing notable pathological changes or affecting their behavior or lifespan. However, the mechanisms by which vector insects evade these viral infections in the brains are poorly understood. In this study, we found that loss of the carbonyl reductase Sniffer (Sni) led to a significant increase in SINV infection in the *Drosophila* brain. Sni regulates reactive oxygen species (ROS) levels, and its depletion leads to elevated ROS, which in turn disrupts the septate junctions (SJs) between subperineurial glia (SPG) cells, compromising the integrity and barrier function of the blood-brain barrier (BBB). Genetic and pharmacological reduction of ROS restored BBB integrity and reduced viral load in the brains of Sni-depleted flies. Additionally, we identified Sni homologs and revealed that the antiviral function of Sni is highly conserved in mosquitoes, where it regulates ROS and protects BBB integrity. Our results revealed an evolutionarily conserved antiviral mechanism in which Sni acts as an antioxidant that protects BBB integrity and restricts viral infection in the vector insect brain.

## Author summary

The mosquito CNS is critical for detecting odors and identifying prey, making it essential for survival and arboviruses transmission. Unlike human, where neurotropic arboviral infections cause severe symptoms, mosquito brains are highly permissive to infection without severe pathology, allowing long-term virus carriage and spread. To identify key antiviral molecules that limit arbovirus CNS infection, we investigated virus-vector insect interactions using SINV-infected *Drosophila* as a model. Through a genetic screen using viral load and survival as readouts, we identified the ROS-regulated carbonyl reductase Sni as an antiviral protein that resists viral infection in the brain. In the absence of Sni, elevated ROS levels disrupted the septate junctions (SJs) and compromised blood-brain barrier (BBB) integrity, leading to increased susceptibility of the brain to neurotropic virus infection. Furthermore, we identified Sni homologs in *Aedes albopictus* and *Aedes aegypti*, demonstrating that the antiviral mechanism of Sni is conserved in mosquitoes, providing new insights and strategies to control the spread of arboviral diseases.

## Introduction

Mosquitoes are natural vectors for arthropod-borne viruses (arboviruses) and transmit them to human hosts through bites, which often results in severe diseases that cause symptoms such as fever, rash, arthritis, and encephalitis in humans [1–6]. However, effective treatments for these arboviral diseases remain limited. Various arboviruses transmitted by mosquitoes, including Sindbis virus (SINV), Zika virus (ZIKV), West Nile virus (WNV), and Japanese encephalitis virus (JEV), can infect the human brain and cause long-term neurological damage [7–12]. Among these, SINV is the prototype virus of the alphavirus genus and has been widely used to study the pathogenesis of encephalitis transmitted by arthropods [13–17]. The central nervous system (CNS) of mosquitoes plays a crucial role in odor recognition and prey detection and, thus, is essential for virus transmission [18,19]. Unlike human brains, where arboviral infections result in severe neurological symptoms, mosquito brains do not exhibit significant pathological changes upon infection, nor do these infections affect the behavior or lifespan of mosquitoes, allowing them to effectively carry and transmit the virus for long periods of time [20,21]. This indicates that insect brains have developed specific immune mechanisms to limit viral infection, thereby protecting tissues from viral damage [22–26]. In particular, an *Aedes aegypti* homolog of the neural factor Hig can prevent lethal flavivirus infections in the CNS of mosquitoes [19]. Due to their sophisticated genetic tools and susceptibility to genetic manipulation, *Drosophila* are an invaluable model for studying innate immunity against various arboviruses, including SINV, JEV, Dengue, and WNV [25–32]. Our previous work has shown that inflammation and dSTING-mediated autophagy restrict ZIKV infection in *Drosophila* brains [33,34]. However, it remains unclear how vector insects prevent arboviruses from crossing the blood-brain barrier (BBB) and infecting the brain.

Reactive oxygen species (ROS), including superoxide anion radicals, hydrogen peroxide, hydroxyl radicals, and singlet oxygen, are cellular metabolic byproducts that play dual roles in biological systems, acting both as potential signaling molecules and as sources of oxidative damage [35–37]. Maintaining the balance between the generation and clearance of ROS is vital for cellular homeostasis and preventing oxidative damage in tissues [37–41]. For example, excessive ROS concentrations in the hemolymph of *Drosophila* disrupt Malpighian tubule excretory function, leading to increased susceptibility to both infection and tissue damage [42]. Cells use specialized enzymatic systems to maintain ROS balance, where NADPH oxidases, including Nox and Duox, generate superoxide anions [43,44], and antioxidant enzymes like superoxide dismutase (SOD) and catalase (Cat) mitigate ROS damage by converting superoxide anions into hydrogen peroxide, which is subsequently broken down [45–47]. The brain consumes a substantial amount of oxygen, almost entirely relies on oxidative phosphorylation for energy, and contains high concentrations of easily peroxidizable lipids, making it particularly susceptible to oxidative damage [48–51]. Excess ROS can lead to significant neuronal damage, which contributes to CNS pathologies and neurodegenerative disorders, such as Alzheimer’s, Parkinson’s, and Huntington’s diseases [52–54]. In *Drosophila*, elevated neuronal ROS levels due to mitochondrial defects activates signaling pathways that induce the accumulation of lipid droplets and lipid peroxidation in glial cells, ultimately leading to neurodegeneration [55–57].

Similar to that in mammals, the BBB in arthropods is formed of glial cells, specifically subperineurial glia (SPG) and perineurial glia (PG) [58–60]. This barrier serves as a crucial line of defense, protecting the CNS from toxins and pathogens [61,62]. The SPG and their septate junctions (SJs), which consist of a network of SJ proteins, such as Neurexin IV (NrxIV), Neuroglian (Nrg), and Discs large (Dlg1), form a tight diffusion barrier encircling the CNS [63–65]. Disruption of these components leads to a compromised, porous BBB [58,63,66]. In mammals, several mechanisms regulate these junction proteins to maintain the integrity of the BBB and prevent neurotropic viruses from infecting the brain [67]. For example, the TAM receptors Axl and Mertk support BBB integrity during viral infections by tightening the junctions of brain microvascular endothelial cells [68]. Mice lacking Axl and Mertk exhibit significantly increased BBB permeability, making it easier for viruses to penetrate the CNS. Consequently, these mice are more susceptible to neuroinvasive viruses such as WNV and La Crosse encephalitis viruses, showing higher mortality rates and increased viral loads in the brain [68]. Additionally, IFN-λ exerts antiviral effects in the brain not through directly inhibiting viral replication but through enhancing the integrity of the BBB via the regulation of two key tight junction proteins, ZO-1 (homologous to *Drosophila* Dlg1) and Claudin-5 [69]. These proteins strengthen the tight junctions of brain microvascular endothelial cells and limit the invasion of WNV into the CNS [69]. However, the mechanisms by which vector mosquitoes maintain BBB integrity to restrict arboviral brain infection require further exploration.

In this study, we sought to identify the specific antiviral mechanisms that limit viral infection in the insect brain. Through a candidate genetic screen in *Drosophila* for SINV infection, we identified the short-chain dehydrogenase/reductase (SDR) family member Sni as a critical antiviral factor in the brain. Loss of Sni expression in *Drosophila* led to increased ROS levels, disruption of the SJ structure, and increased BBB permeability, thereby facilitating neurotropic SINV entry and replication in the brain. Furthermore, Sni function was conserved in mosquitoes. Its knockdown increased BBB permeability in mosquitoes and facilitated SINV brain infection. These findings provide profound insights into the evolutionarily conserved antiviral mechanisms of vector insects against arboviruses infection in the brain.

## Results

### Candidate genetic screening identified Sni as an antiviral factor in *Drosophila*

**Fig 1:**
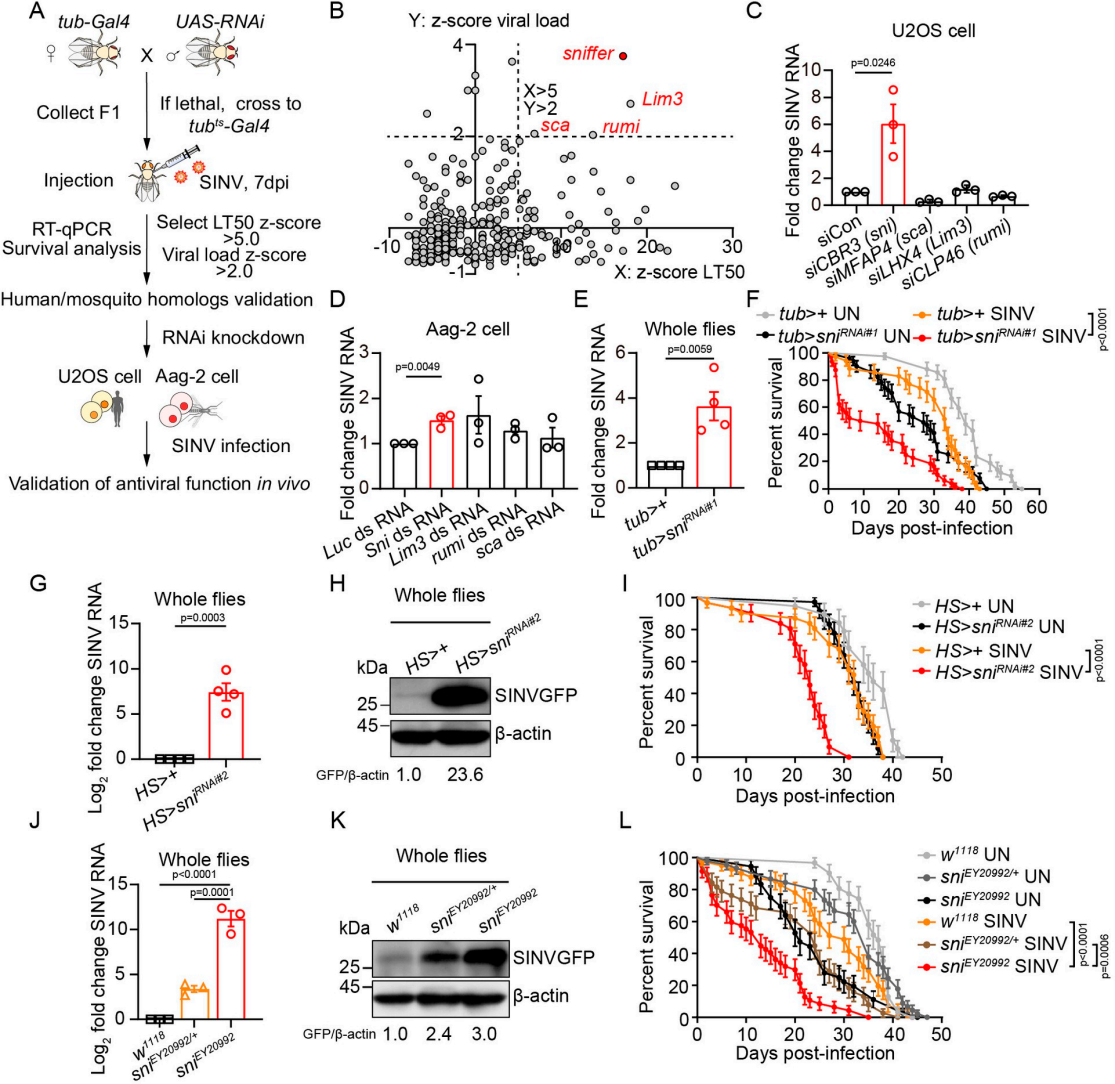
Candidate genetic screening identified Sni as an antiviral factor. (A) Schematic for screen setup. SINV was injected into 4-7-day-old F1 flies. (B) Z-score distributions of the screen results. Genes with median lethal time (LT50) z-score >5.0 and viral load z-score >2.0 were selected for further study. (C and D) RT-qPCR analysis of SINV viral load in human U2OS cells (C) and mosquito Aag-2 cells (D) treated with siRNA/dsRNA targeting specific genes for 48 hours, followed by SINV infection for 24 hours. (E) RT-qPCR analysis of SINV viral load in SINV-infected control (*tub>+*) or *sni* RNAi (*tub>sni*^*RNAi#1*^) flies at 7 days post infection (dpi). (F) Percent survival of control (*tub>+*) or *sni* RNAi (*tub>sni*^*RNAi#1*^) flies with or without SINV infection. The numbers of quantified flies: 42 (*tub>+* UN), 51 (*tub>sni*^*RNAi#1*^ UN), 46 (*tub>+* SINV), and 48 (*tub>sni*^*RNAi#1*^ SINV). (G) RT-qPCR analysis of SINV viral load in SINV-infected *sni* RNAi (*HS>sni*^*RNAi#2*^) or control (*HS>+*) whole flies at 7 dpi. (H) Western blot analysis of SINV viral load in SINV-infected *sni* RNAi (*HS>sni*^*RNAi#2*^) or control (*HS>+*) whole flies at 7 dpi. β-actin was used as a loading control. (I) Percent survival of *sni* RNAi (*HS>sni*^*RNAi#2*^) or control (*HS>+*) flies with or without SINV infection. The numbers of quantified flies: 38 (*HS>+* UN), 36 (*HS>sni*^*RNAi#2*^ UN), 31 (*HS>+* SINV), and 31 (*HS>sni*^*RNAi#2*^ SINV). (J) RT-qPCR analysis of SINV viral load in SINV-infected *sni* mutant (*sni*^*EY20992*^) or control (*w*^*1118*^ and *sni*^*EY20992/+*^) whole flies at 7 dpi. (K) Western blot analysis of SINV viral load in SINV-infected *sni* mutant (*sni*^*EY20992*^) or control (*w*^*1118*^ and *sni*^*EY20992/+*^) whole flies at 7 dpi. β-actin was used as a loading control. (L) Percent survival of *w*^*1118*^, *sni*^*EY20992/+*^ or *sni*^*EY20992*^ flies with or without SINV infection. The numbers of quantified flies: 45 (*w*^*1118*^ UN), 51 (*sni*^*EY20992/+*^ UN), 48 (*sni*^*EY20992*^ UN), 41 (*w*^*1118*^ SINV), 52 (*sni*^*EY20992/+*^ SINV), and 47 (*sni*^*EY20992*^ SINV). Data represent mean ± SEM. Statistical analysis was performed using two-tailed unpaired Student’s t test (C, D, E and G), One-way ANOVA (J) and Log-Rank test (F, I and L). At least three independent experiments were performed.

To identify novel antiviral factors in insects, we selected SINV as a model arbovirus to infect *Drosophila* and conducted a candidate gene screening. We selected candidate genes based on several assumptions: antiviral immune factors in the brain likely function by binding to viral RNA or proteins [70–72]; viral infections are closely associated with brain aging [73–75]; secreted proteins, such as neuropeptides, typically serve as signaling molecules to regulate immune responses [76–78]; and potent antiviral effectors may be induced by viral infection [29,32]. In particular, we selected five groups of candidates: RNA-binding proteins, protein modification enzymes, aging-related genes, secreted proteins, and genes induced by SINV infection (S1 Table). Using the Gal4/UAS system for RNAi knockdown, we screened 295 candidate genes. In addition to the SINV viral load, we recorded the survival curves as another screening readout (Fig 1A). The median lethality time (LT50) and viral load z-scores were calculated, with cutoff values set at LT50 z-score > 5.0 and viral load z-score > 2.0. Using these screening criteria, we identified four genes: *sni*, *Lim3*, *sca*, and *rumi* (Fig 1B and S1 Table). To explore the conservation of these antiviral genes among human hosts and mosquito vectors, we knocked down the corresponding homologous genes using siRNA and dsRNA, and infected human U2OS and mosquito Aag-2 cells with SINV (Figs 1A, S1A and S1B). In Aag-2 and U2OS cells, knockdown of the mosquito and human homologs of Sni resulted in increased viral loads, respectively (p<0.05; Fig 1C and 1D). The *sni* gene encodes a homodimeric carbonyl reductase of the SDR family that catalyzes the reduction of the lipid-derived aldehyde 4-HNE [79–81], thereby contributing to the prevention of oxidative stress-induced neurodegeneration and cell apoptosis [82]. In addition to their higher viral load, *sni-*knockdown flies were also more susceptible to SINV infection and succumbed to infection more quickly than controls (p<0.05; Figs 1E, 1F and S1C).

To rule out potential off-target effects of RNAi, we used a second RNAi *sni*^*RNAi#2*^ and *sni*^*EY20992*^ mutant strains to further validate its antiviral phenotype. Consistent with the *sni*^*RNAi#1*^ result, the *sni*^*RNAi#2*^ knockdown flies showed increased viral replication and a significant reduction in survival (p<0.01; Figs 1G–1I and S1D). The *sni*^*EY20992*^ strain had a P-element insertion in the 5’ UTR of the *sni* gene and *sni* mRNA levels were reduced by 36.1% in homozygous mutants compared to those in heterozygous controls (p<0.001; S1E and S1F Fig). Although the P element was also located within an intron of the *Trxr1* gene, RT-qPCR results indicated that it did not affect *Trxr1* mRNA expression levels (S1E and S1G Fig). Similar to the phenotype of *sni* null mutant [82], the *sni*^*EY20992*^ mutation led to notable neurodegeneration and a reduced lifespan under oxidative stress (p<0.0001; S1H–S1J Fig), confirming its effectiveness in disrupting Sni function. *sni*^*EY20992*^ homozygous flies exhibited higher viral loads and decreased survival, indicating increased susceptibility to SINV infection compared to that of the heterozygous controls (p<0.001; Fig 1J–1L). Furthermore, we assessed the locomotor ability of *sni*^*EY20992*^ homozygous flies in comparison to age-matched controls, both with and without SINV infection, using a climbing assay (S1K Fig). We found that *sni*^*EY20992*^ flies exhibited reduced climbing ability relative to age-matched controls at all time points, and this impairment worsened with age (p<0.01; S1K Fig). Additionally, the climbing defects in *sni* mutants were exacerbated by SINV infection, as 4-week-old SINV-infected mutant flies demonstrated a significant decrease in climbing ability compared to uninfected mutants of the same age (p<0.01; S1K Fig). Taken together, these results suggested that the carbonyl reductase Sni restricts SINV infection, and its antiviral function is highly conserved in human and mosquito.

### Sni restricts SINV infection in the brains and intestines of adult *Drosophila*

**Fig 2:**
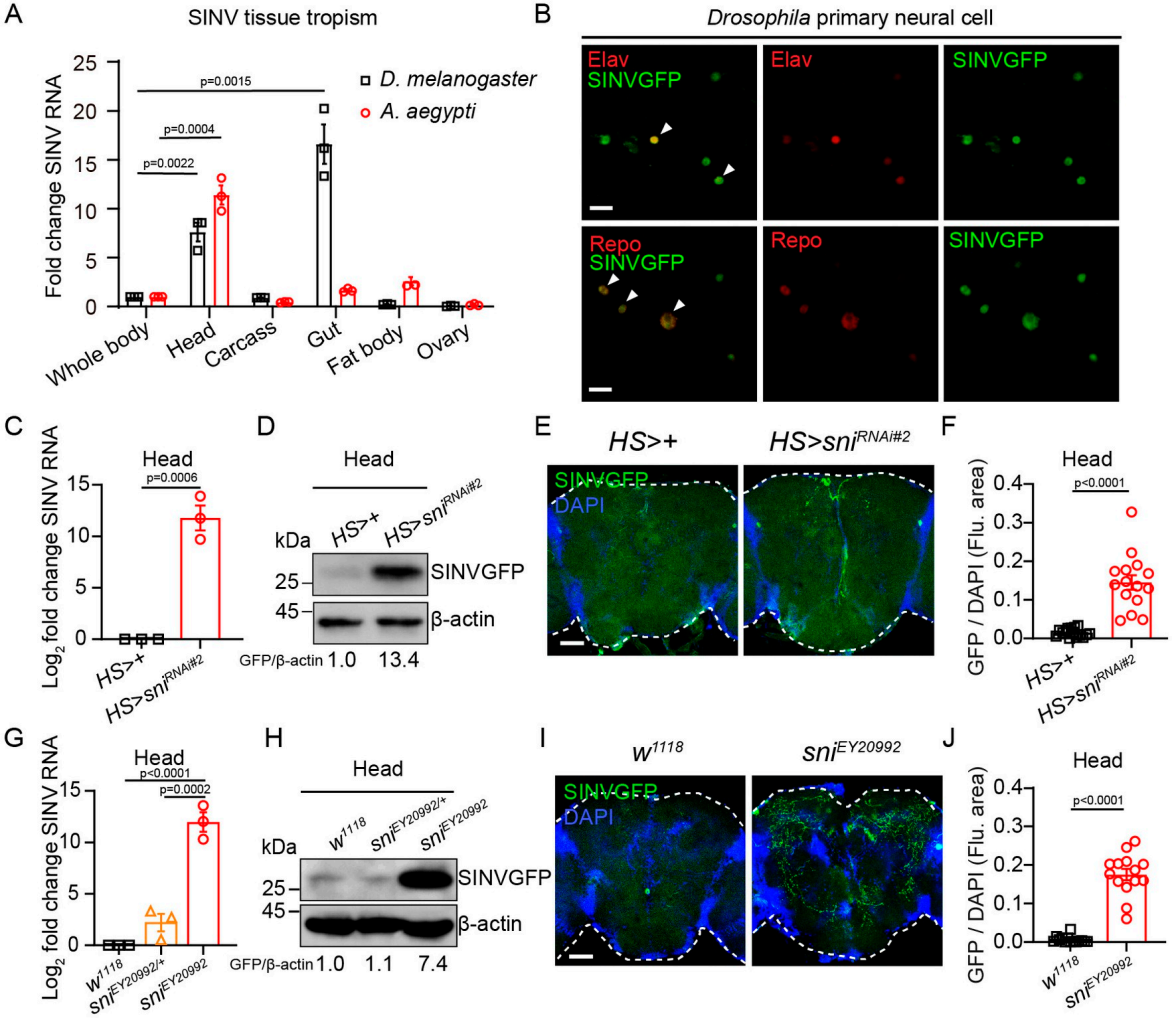
Sni restricts SINV infection in the brains of adult *Drosophila*. (A) RT-qPCR analysis of SINV tissue tropism in *D*. *melanogaster* at 7dpi and *A*. *aegypti* at 3dpi. (B) Representative immunofluorescence images of *Drosophila* primary neural cells stained for SINVGFP and either Elav or Repo markers at 24 hours post-infection with SINV (virus, green; Elav or Repo, red). Arrowheads indicate co-localization of SINVGFP with Elav or Repo. (C) RT-qPCR analysis of SINV viral load in SINV-infected *sni* RNAi (*HS>sni*^*RNAi#2*^) or control (*HS>+*) fly heads at 7 dpi. (D) Western blot analysis of SINV viral load in SINV-infected *sni* RNAi (*HS>sni*^*RNAi#2*^) or control (*HS>+*) fly heads at 7 dpi. β-actin was used as a loading control. (E) Representative immunofluorescence images of heads stained for SINVGFP in SINV-infected control (*HS>+*) or *sni* RNAi (*HS>sni*^*RNAi#2*^) flies at 7 dpi (virus, green; nuclei, blue). (F) Quantification of SINVGFP fluorescence area in the experiments (E). The numbers of quantified *Drosophila* heads: 15 (*HS>+*) and 15 (*HS>sni*^*RNAi#2*^). (G) RT-qPCR analysis of SINV viral load in SINV-infected *sni* mutant (*sni*^*EY20992*^) or control (*w*^*1118*^ and *sni*^*EY20992/+*^) fly heads at 7 dpi. (H) Western blot analysis of SINV viral load in SINV-infected *sni* mutant (*sni*^*EY20992*^) or control (*w*^*1118*^ and *sni*^*EY20992/+*^) fly heads at 7 dpi. β-actin was used as a loading control. (I) Representative immunofluorescence images of heads stained for SINVGFP in SINV-infected control (*w*^*1118*^) or *sni* mutant (*sni*^*EY20992*^) flies at 7 dpi (virus, green; nuclei, blue). (J) Quantification of SINVGFP fluorescence area in (I). The numbers of quantified *Drosophila* heads: 14 (*w*^*1118*^) and 14 (*sni*^*EY20992*^). Data represent mean ± SEM. In (E and I), the dotted line represents the edge of the *Drosophila* head. Scale bars represent 10 μm (B) and 50 μm (E and I). Statistical analysis was performed using two-tailed unpaired Student’s t-test (A, C, F, and J) and One-way ANOVA (G). At least three independent experiments were performed.

In mammals, the SINV virus can target the brain and establish severe infection, making SINV-infected mice a model for studying the pathogenesis of alphavirus-induced encephalitis [16,17]. To investigate the tissue tropism of SINV in insects, we measured viral loads in various tissues from SINV-infected wild-type *Drosophila* and mosquitoes using RT-qPCR. Compared to whole-body infection levels, viral loads were significantly elevated in the heads of both *Drosophila* (7.6 ± 1.3-fold, p = 0.0022) and mosquitoes (11.4 ± 1.4-fold, p = 0.0004), with robust infection also detected in the intestines of *Drosophila* (16.6 ± 2.8-fold, p = 0.0015) (Fig 2A). Additionally, in primary cells derived from the *Drosophila* larval brain, SINVGFP colocalized with the neuronal marker Elav and the glial marker Repo, indicating that SINV can infect both neurons and glial cells (Fig 2B). Next, we set out to determine whether Sni is antiviral in the brain. We examined the brains of SINV-infected *sni* RNAi and *sni* mutant flies using RT-qPCR (Figs 2C, 2G and S2A), western blots (Fig 2D and 2H) and immunofluorescence (Figs 2E, 2I and S2B). Our results showed a significant increase in SINV infection in both groups compared to controls (p<0.05; Figs 2C–2J and S2A–S2C). Furthermore, immunofluorescence revealed an increase in SINV infection foci in the brains of *sni* RNAi and *sni* mutant flies compared to that in the controls, indicating active viral replication (Figs 2E, 2F, 2I, 2J, S2B and S2C). Additionally, in the brain of *sni* mutant flies, SINVGFP was colocalized with both the neuronal marker Elav and the glial marker Repo (S2D Fig). Moreover, infection and breaching of the mosquito gut barrier is a key step of mosquito-borne viral transmission [83]. We also observed increased SINV infection in the intestines of *sni* RNAi and *sni* mutant flies compared to controls (p<0.0001; S2E–S2L Fig). Collectively, these results suggest that Sni restricts SINV infection in the brains and intestines of *Drosophila*.

### Sni restricts viral infections by modulating ROS levels in the brain

**Fig 3:**
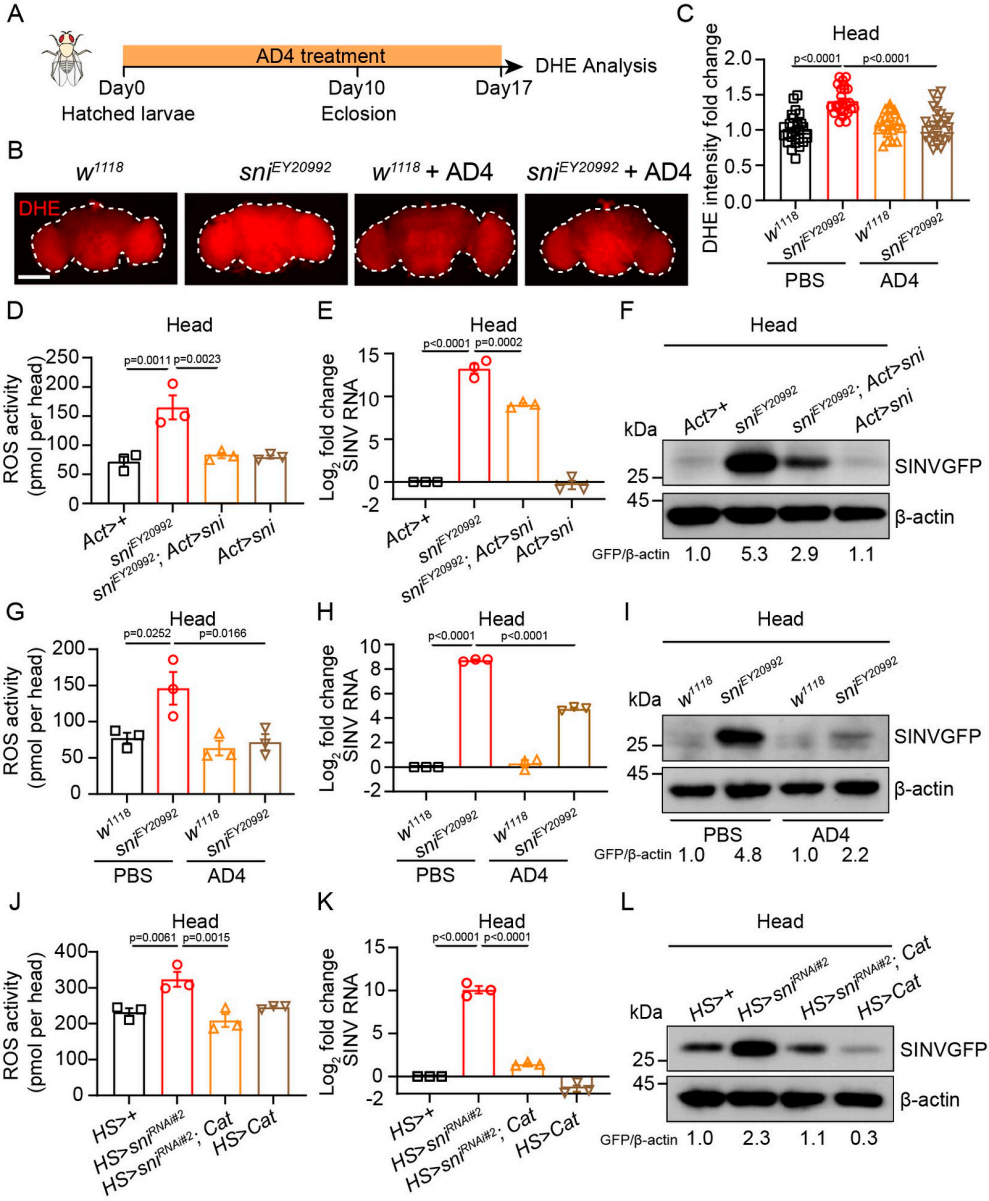
Sni restricts viral infection by modulating ROS levels in the brain. (A) Schematic timeline of AD4 treatment and DHE assay in (B). (B) Representative immunofluorescence images of adult heads stained for DHE in control (*w*^*1118*^) or *sni* mutant (*sni*^*EY20992*^) flies after feeding with 40 μg/ml AD4 or PBS (DHE, red). (C) Quantification of DHE intensity in (B). *w*^*1118*^ flies fed with PBS were used as control. The numbers of quantified brains from left to right are 27, 25, 21, and 24. (D) ROS activity measured by H_2_O_2_ assay in 25 pooled adult heads of control (*Act>+*), *sni* mutant (*sni*^*EY20992*^), *sni* rescue (*sni*^*EY20992*^; *Act>sni*) and *sni* overexpression (*Act>sni*) flies. (E) RT-qPCR analysis of SINV viral load in adult heads of SINV-infected control (*Act>+*), *sni* mutant (*sni*^*EY20992*^), *sni* rescue (*sni*^*EY20992*^; *Act>sni*) and *sni* overexpression (*Act>sni*) flies. (F) Western blot analysis of SINV viral load in adult heads of SINV-infected control (*Act>+*), *sni* mutant (*sni*^*EY20992*^), *sni* rescue (*sni*^*EY20992*^; *Act>sni*) and *sni* overexpression (*Act>sni*) flies at 7 dpi. β-actin was used as a loading control. (G) ROS activity measured by H_2_O_2_ assay in 25 pooled adult heads of control (*w*^*1118*^) or *sni* mutant (*sni*^*EY20992*^) flies with PBS or AD4 feeding. (H) RT-qPCR analysis of SINV viral load in adult heads of SINV-infected *sni* mutant (*sni*^*EY20992*^) or control (*w*^*1118*^) flies with PBS or AD4 feeding at 7 dpi. (I) Western blot analysis of SINV viral load in adult heads of SINV-infected *sni* mutant (*sni*^*EY20992*^) or control (*w*^*1118*^) flies with PBS or AD4 feeding at 7 dpi. β-actin was used as a loading control. (J) ROS activity measured by H_2_O_2_ assay in 25 pooled adult heads of control (*HS>+*), *sni* RNAi (*HS>sni*^*RNAi#2*^), *Cat* rescue (*HS>sni*^*RNAi#2*^; *Cat*) and *Cat* overexpression (*HS>Cat*) flies. (K) RT-qPCR analysis of SINV viral load in adult heads of SINV-infected control (*HS>+*), *sni* RNAi (*HS>sni*^*RNAi#2*^), *Cat* rescue (*HS>sni*^*RNAi#2*^; *Cat*) and *Cat* overexpression (*HS>Cat*) flies at 7 dpi. (L) Western blot analysis of SINV viral load in adult heads of SINV-infected control (*HS>+*), *sni* RNAi (*HS>sni*^*RNAi#2*^), *Cat* rescue (*HS>sni*^*RNAi#2*^; *Cat*) and *Cat* overexpression (*HS>Cat*) flies at 7 dpi. β-actin was used as a loading control. Data represent mean ± SEM. Scale bars represent 100 μm (B). In (B), the dotted line represents the edge of the *Drosophila* brain. Statistical analysis was performed using One-way ANOVA (C–E, G, H, J and K). At least three independent experiments were performed.

As a carbonyl reductase, Sni converts deleterious aldehydic and keto byproducts into less reactive aldehydes, thereby mitigating oxidative stress [81,82]. We hypothesized that the depletion of *sni* expression leads to increased ROS levels, subsequently resulting in increased brain tissue damage and susceptibility to SINV. To test this hypothesis, we performed dihydroethidium (DHE) staining and an H_2_O_2_ assay to measure ROS levels in the brains of larval and adult *sni* RNAi and mutant flies. We observed that ROS levels were significantly higher in the brains of *sni* RNAi and mutant flies compared to control (p<0.01; Figs 3A–3D, 3G, 3J and S3A–S3G). However, administering N-acetyl cysteine amide (AD4), a BBB penetrating antioxidant, or overexpressing the *Cat* gene in these flies effectively counteracted the increase in ROS levels in the brains of *sni*-deficient flies (Figs 3A–3C, 3G, 3J and S3A–S3D). To confirm that the increases in ROS levels and viral load were due to the loss of Sni, we generated a UAS-Sni-Flag *Drosophila* line for Sni overexpression and validated its expression by western blotting (S3H Fig). We found that Sni overexpression rescued the elevated ROS levels (*sni*^*EY20992*^, 164.8 ± 29.1 pmol per head vs *sni*^*EY20992*^*; Act>sni* 82.2 ± 6.5 pmol per head; p = 0.0023; Fig 3D), and the increased viral load caused by Sni deficiency (p<0.01; Fig 3E and 3F). To determine whether ROS-mediated damage was the reason for increased viral susceptibility, we restored ROS levels in these flies and monitored the SINV viral load in their brains. We found that both genetic and pharmacological restoration of ROS levels significantly reduced the SINV viral load in the brains of *sni*-deficient flies (p<0.0001; Fig 3H, 3I, 3K and 3L). Collectively, these results suggest that Sni restricts brain viral infections by suppressing ROS production.

### Elevated ROS disrupts BBB integrity in the brains of *sni*-deficient flies

**Fig 4:**
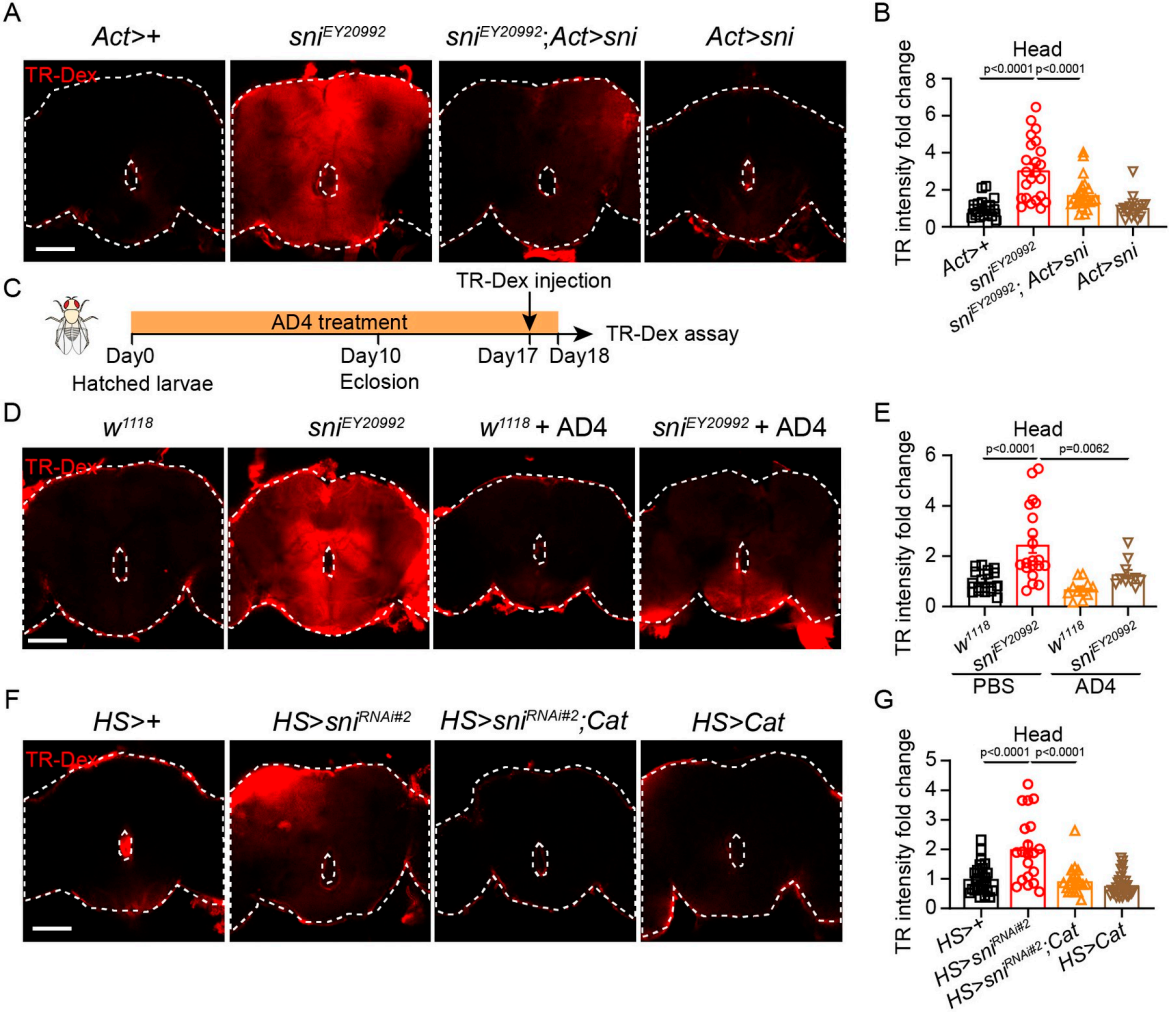
The depletion of *sni* led to ROS-induced disruption of the BBB integrity in the brain. (A) Representative immunofluorescence images of adult heads stained for TR-Dex in control (*Act>+*), *sni* mutant (*sni*^*EY20992*^), *sni* rescue (*sni*^*EY20992*^; *Act>sni*) and *sni* overexpression (*Act>sni*) flies. (B) Quantification of TR-Dex intensity in (A). *Act>+* flies were used as controls. The numbers of quantified *Drosophila* heads:21 (*Act>+*), 23(*sni*^*EY20992*^), 24 (*sni*^*EY20992*^; *Act>sni*) and 17 (*Act>sni*). (C) Schematic timeline of AD4 treatment and TR-Dex penetration assay in (D). (D) Representative immunofluorescence images of adult heads stained for TR-Dex in control (*w*^*1118*^) or *sni* mutant (*sni*^*EY20992*^) flies fed with PBS or AD4 (TR-Dex, red). (E) Quantification of TR-Dex intensity in (D). *w*^*1118*^ flies fed with PBS were used as controls. The numbers of quantified *Drosophila* heads: 18 (*w*^*1118*^+PBS), 20 (*sni*^*EY20992*^+PBS), 11 (*w*^*1118*^+AD4), and 10 (*sni*^*EY20992*^+AD4). (F) Representative immunofluorescence images of adult heads stained for TR-Dex in control (*HS>+*), *sni* RNAi (*HS>sni*^*RNAi#2*^), *Cat* rescue *HS>sni*^*RNAi#2*^; *Cat*) and *Cat* overexpression (*HS>Cat*) flies (TR-Dex, red). (G) Quantification of TR-Dex intensity in (F). *HS>+* flies were used as controls. The numbers of quantified *Drosophila* heads: 28 (*HS>+*), 20 (*HS>sni*^*RNAi#2*^), 21 (*HS>sni*^*RNAi#2*^; *Cat*), and 30 (*HS>Cat*). Data represent mean ± SEM. Scale bars represent 50 μm (A, D and F). In (A, D and F), the dotted line represents the edge of the *Drosophila* head. Statistical analysis was performed using One-way ANOVA (B, E and G). At least three independent experiments were performed.

We speculated that increased ROS production in *sni*-depleted brains attacks key BBB components, compromising its structural integrity and allowing SINV invasion. To verify this, we injected the body cavities of both mutant and control flies with TR-Dex, a fluorescent dye commonly used to assess BBB integrity [84], and used confocal imaging to assess its uptake into the brain. We observed a significant accumulation of dye in the brains of *sni* mutant (3.1 ± 1.6-fold, p<0.0001; Fig 4A and 4B) and *sni* RNAi flies (2.0 ± 1.1-fold, p<0.0001; Fig 4F and 4G), indicating altered BBB permeability and disrupted function (p<0.01; Figs 4A–4G, S4A and S4B). However, Sni overexpression successfully attenuated the dye accumulation phenotype of *sni* mutant (Fig 4A and 4B). Sod2 and Cat are key antioxidant enzymes that mitigate oxidative stress in the *Drosophila* brain [85–87]. As a control, knocking down *Sod2* led to the accumulation of Tr-Dex and an increase in viral load in the brain (p<0.001; S4C–S4E Fig). Notably, Tr-Dex accumulation was not observed in *sni*-deficient flies treated with the antioxidant AD4 or in those overexpressing *Cat* (Fig 4C–4G). Collectively, these results suggest that the depletion of *sni* expression leads to increased permeability and functional impairment of the BBB, whereas genetic and pharmacological downregulation of ROS levels can restore BBB functionality.

### The depletion of *sni* led to disrupted SJ structure within the BBB

**Fig 5:**
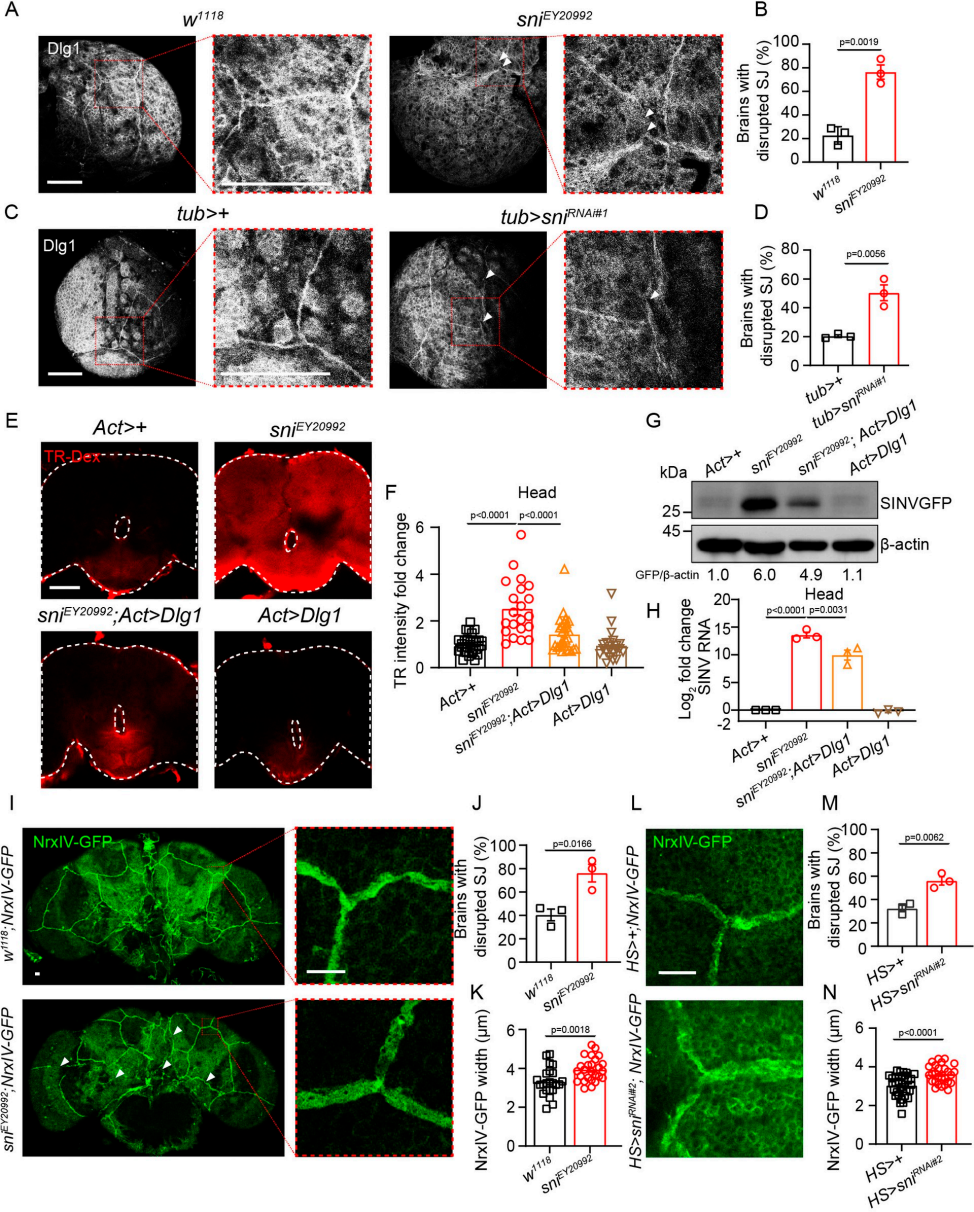
The depletion of *sni* led to disrupted SJ integrity in the BBB. (A and C) Representative immunofluorescence images of L3 larval brains stained for Dlg1 in (A) control (*w*^*1118*^) and *sni* mutant (*sni*^*EY20992*^) flies and (C) control (*tub>+*) and *sni* RNAi (*tub>sni*^*RNAi#1*^) flies (Dlg1, white). Arrowheads indicate regions with disrupted SJ belts. (B and D) Quantification of the percentage of larval brains with disrupted SJ belts as shown in (A) and (C). The numbers of quantified *Drosophila* brains: (B) 22 (*w*^*1118*^) and 22 (*sni*^*EY20992*^); (D) 35 (*tub>+*) and 46 (*tub>sni*^*RNAi#1*^). (E) Representative immunofluorescence images of adult heads stained for TR-Dex in control (*Act>+*), *sni* mutant (*sni*^*EY20992*^), *Dlg1* rescue (*sni*^*EY20992*^; *Act>Dlg1*) and *Dlg1* overexpression (*Act>Dlg1*) flies. (F) Quantification of TR-Dex intensity in (E). *Act>+* flies were used as controls. The numbers of quantified *Drosophila* heads:29 (*Act>+*), 21(*sni*^*EY20992*^), 28 (*sni*^*EY20992*^; *Act>Dlg1*) and 21 (*Act>Dlg1*). (G) Western blot analysis of SINV viral load in adult heads of SINV-infected control (*Act>+*), *sni* mutant (*sni*^*EY20992*^), *Dlg1* rescue (*sni*^*EY20992*^; *Act>Dlg1*) and *Dlg1* overexpression (*Act>Dlg1*) flies at 7 dpi. β-actin was used as a loading control. (H) RT-qPCR analysis of SINV viral load in adult heads of SINV-infected control (*Act>+*), *sni* mutant (*sni*^*EY20992*^), *Dlg1* rescue (*sni*^*EY20992*^; *Act>Dlg1*) and *Dlg1* overexpression (*Act>Dlg1*) flies. (I and L) Representative immunofluorescence images of adult heads stained for NrxIV-GFP in (I) control (*w*^*1118*^) and *sni* mutant (*sni*^*EY20992*^) flies, and (L) control (*HS>+*) and *sni* RNAi (*HS>sni*^*RNAi#2*^) flies. Arrowheads indicate disrupted SJ belts. (J, K, M, and N) Quantification of (J and M) the percentage of adult heads with disrupted SJ belts, and (K and N) NrxIV-GFP width as shown in (I and L). *w*^*1118*^ and *HS>+* flies were used as controls. The numbers of quantified *Drosophila* heads: (J) 56 (*w*^*1118*^) and 45(*sni*^*EY20992*^); (K) 23 (*w*^*1118*^) and 26(*sni*^*EY20992*^); (M) 34 (*HS>+*) and 27 (*HS>sni*^*RNAi#2*^) and (N) 34 (*HS>+*) and 27 (*HS>sni*^*RNAi#2*^). Data represent mean ± SEM. Scale bars represent 50 μm (A, C and E) and 10 μm (I and L). In (E), the dotted line represents the edge of the *Drosophila* head. Statistical analysis was performed using two-tailed unpaired Student’s t-test (B, D, J, K, M and N) and One-way ANOVA (F and H). At least three independent experiments were performed.

SJs in the BBB of insects like *Drosophila*, homologous to tight junctions in vertebrates, connect glial cells to form a barrier that prevents pathogens from entering the nervous system [63]. As a control to evaluate whether BBB structural damage contributes to the increased SINV viral load in the brain, we specifically knocked down *moody* and *NrxIV* in the SPG using *moody-gal4*, which mimics BBB structural damage and then infected the flies with SINV. The depletion of these key SJ components led to a significant increase in SINV viral load in the brain (p<0.01; S5A Fig). To investigate the molecular mechanism by which *sni* deficiency leads to elevated ROS levels and subsequent BBB damage, we used Dlg1, a key SJ protein, as a marker to assess the structural integrity of SJs. Dlg1 colocalized with *moody-Gal4>GFP* (S5B Fig), indicating that SJs are distributed in the SPG layer of the BBB [88]. Consistent with this, when we knocked down *sni* expression in different brain cell types, the highest viral load was observed in brains where *sni* was knocked down using *repo-Gal4* and *moody-Gal4*, suggesting that Sni primarily functions in the SPG (p<0.0001; S5C Fig). Immunostaining for Dlg1 revealed that the proportion of larval brains exhibiting disrupted SJ belts was significantly higher in *sni* mutants and RNAi knockdown flies than in wild-type flies (p<0.01; Fig 5A–5D). Specifically, disruptions of SJs were observed in 76.4% of brain samples from *sni* mutant flies versus 22.4% in control flies (p = 0.0019; Fig 5A and 5B). Similarly, in larval brains of *sni* RNAi flies, 50.4% displayed SJ defects compared to 20.3% in control larval brains (p = 0.0056; Fig 5C and 5D). This phenotype resembles the disrupted SJ structure observed in the brain of *miR-285* mutant flies [88] and the tight junction discontinuities seen in the brains of *Axl−/− Mertk−/−* or *Ifnlr1−/−* mice [68,69], suggesting that Sni plays an essential role in maintaining BBB integrity, similar to the functions of these genes. Additionally, the Dlg1 protein expression level in *sni* mutant brains was reduced by 20% compared to that in controls (S5D Fig). Given that *sni* depletion impairs Dlg1 expression, we sought to determine whether Dlg1 overexpression could rescue the BBB permeability defects in the *sni* mutant. Indeed, following Dlg1 overexpression, we observed a reduction in Tr-Dex dye leakage into the brain and a corresponding decrease in viral load compared to the *sni* mutant alone (p<0.01; Fig 5E–5H). These results suggest that increasing Dlg1 expression can partially restore BBB integrity compromised by *sni* depletion, thus limiting viral invasion into the brain. To further characterize the structural changes in SJs caused by *sni* depletion, we used NrxIV-GFP and Nrg-GFP reporters to label SJs in the adult *Drosophila* brain. Compared to the control group, *sni* mutants and RNAi knockdown flies showed a significantly higher proportion of brains with NrxIV-GFP-labeled SJ belts exhibiting more frequent breaks, along with a more diffuse and widened appearance (p<0.05; Fig 5I–5N), a phenotype similar to that previously reported in *moody* mutant flies [63]. The average width of NrxIV-GFP-labeled SJ belts in *sni* mutants (3.93 ± 0.60 μm) showed a significant increase of 0.62 μm compared to *w*^*1118*^ flies (3.31 ± 0.70 μm; p = 0.0018; Fig 5K). Similarly, *sni* RNAi flies showed a wider NrxIV-GFP-labeled SJ belts (3.61 ± 0.47 μm), with an average increase of 0.58 μm compared to *HS>+* flies (3.03 ± 0.54 μm; p<0.0001; Fig 5N). Consistent with these findings, Nrg-GFP-labeled SJ belts in *sni* RNAi flies were also wider and more diffuse (*sni* RNAi 3.41 ± 0.51 μm versus *HS>+* flies 2.75 ± 0.47 μm; p<0.0001; S5E and S5F Fig). Taken together, these results suggested that *sni* deficiency led to elevated ROS levels, which disrupted SJ structure and compromised BBB integrity, consequently facilitating viral infection in the brain.

### The mosquito Sni homolog protects BBB integrity against SINV infection by suppressing ROS

**Fig 6:**
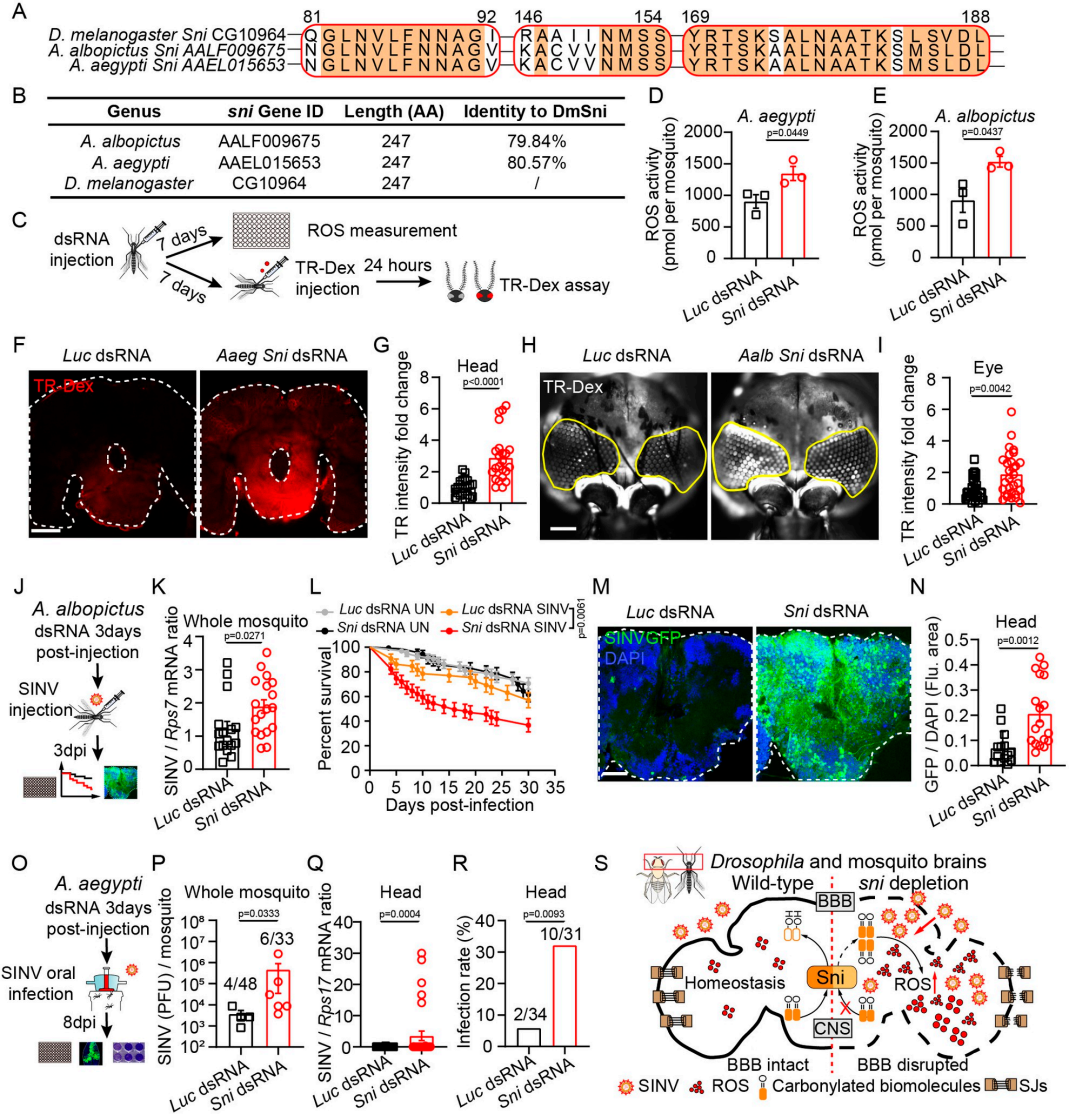
The mosquito Sni homolog protects BBB integrity against SINV infection by suppressing ROS. (A) Alignment of the SDR family functional domains in *Drosophila* Sni compared with homologous proteins AALF009675 and AAEL015653 from *A*. *albopictus* and *A*. *aegypti*. (B) Percentage identity of amino acids between *Drosophila* and mosquito Sni homologs. (C) Experimental scheme of TR-Dex assay and ROS measurement in mosquitoes. (D and E) ROS activity of 6 pooled *Sni* knockdown *A*. *aegypti* (D) and *A*. *albopictus* (E) mosquitoes versus controls (*Luc* dsRNA), measured by H_2_O_2_ assay. (F and G) Representative immunofluorescence images (F) and quantification (G) TR-Dex staining in heads of control (*Luc* dsRNA) and *Sni* knockdown mosquitoes. White dotted line represents the edge of the mosquito head. The numbers of quantified *A*. *aegypti* heads: 22 (*Luc* dsRNA) and 23 (*Aaeg Sni* dsRNA). (H and I) Representative immunofluorescence images (H) and quantification (I) of TR-Dex staining in eyes of controls (*Luc* dsRNA) and *Sni* knockdown mosquitoes. Yellow line represents the edge of the mosquito eye. The numbers of quantified *A*. *albopictus* eyes: 29 (*Luc* dsRNA) and 29 (*Aalb Sni* dsRNA). (J) Experimental scheme of dsRNA and SINV injection in *A*. *albopictus*: dsRNA was injected, followed by SINV injection after 3 days, with viral load assessed by RT-qPCR and immunofluorescence at 3 dpi. (K) RT-qPCR analysis of SINV viral load in SINV infected control (*Luc* dsRNA) or *Sni* knockdown (*Sni* dsRNA) mosquitoes at 3 dpi. Each dot represents an individual mosquito. (L) Percent survival of control (*Luc* dsRNA) and *Sni* knockdown (*Sni* dsRNA) mosquitoes with or without SINV infection. The numbers of quantified mosquitoes: 95 (*Luc* dsRNA UN), 70 (*Sni* dsRNA UN), 65 (*Luc* dsRNA SINV), and 79 (*Sni* dsRNA SINV). (M and N) Representative immunofluorescence images of SINVGFP-stained heads in control (*Luc* dsRNA) and Sni knockdown (*Sni* dsRNA) mosquitoes (M: virus, green; nuclei, blue) and quantification of the SINVGFP fluorescence area (N). The numbers of quantified mosquito heads: 14 (*Luc* dsRNA) and 18 (*Sni* dsRNA). (O) Experimental scheme of SINV oral infection in *A*. *aegypti*. *A*. *aegypti* mosquitoes were injected with *Luc* and *Sni* dsRNA. After 3 days, they were fed blood containing SINV. At 8 dpi, SINV viral load were assessed by plaque assay, RT-qPCR and immunofluorescence. (P) SINV plaque assay for whole-body viral load in SINV-infected control (*Luc* dsRNA) or *Sni* knockdown (*Sni* dsRNA) mosquitoes at 8 dpi. Each dot represents an individual mosquito. The numbers of quantified mosquitoes: 48 (*Luc* dsRNA) and 33 (*Sni* dsRNA). (Q) RT-qPCR analysis of SINV viral load in the heads of SINV infected control (*Luc* dsRNA) or *Sni* knockdown (*Sni* dsRNA) mosquitoes at 8 dpi. Each dot represents an individual mosquito. The numbers of quantified mosquitoes: 34 (*Luc* dsRNA) and 31 (*Sni* dsRNA). (R) Quantification of SINV infection rate for experiments in (Q). (S) Schematic model illustrating how Sni restricts SINV brain infections by regulating ROS and maintaining BBB integrity in *Drosophila* and mosquitos. In wild-type insects, Sni reduces oxidative stress in the brain by converting carbonyl groups in proteins/lipids to hydroxyl groups. Without Sni, carbonyl accumulation leads to increased ROS levels, disrupted SJ structure and reducing the SJ protein Dlg1. This disruption compromises the BBB integrity, allowing neurotropic viruses to penetrate the BBB and proliferate within the CNS. Data represent mean ± SEM. Scale bars represent 50 μm (F and M) and 100 μm (H). Statistical analysis was performed using two-tailed unpaired Student’s t-test (D, E, G, I and N), non-parametric Mann-Whitney test (K, P, and Q), Log-Rank test (L) and Chi-squared test (R). At least three independent experiments were performed.

By performing amino acid sequence alignment, we identified Sni homologs in *Aedes albopictus* (79.84% similarity) and *Aedes aegypti* (80.57% similarity) with highly conserved SDR functional domains (Fig 6A and 6B). We found that the expression of the mosquito *Sni* gene was induced by SINV infection in both C6/36 cells and adult mosquitoes (p<0.001; S6A Fig). As a control experiment, we first examined whether elevated ROS levels and disruption of the BBB would increase SINV viral loads in the mosquito head. Knocking down *Sod2* to elevate ROS levels (p<0.05; S6B and S6C Fig), and disrupting the BBB through *Dlg1* and *NrxIV* knockdown (p<0.05; S6E Fig), resulted in significantly higher SINV loads in the heads of these mosquitoes compared to *Luciferase* knockdown controls (p<0.01; S6D and S6F Fig). Furthermore, TR-Dex dye accumulated more prominently in the eyes of *Dlg1* and *NrxIV* knockdown *A*. *aegypti* mosquitoes, compared to *Luciferase* knockdown controls (p<0.01; S6G and S6H Fig). These results suggest that the mosquito and *Drosophila* BBBs share conserved functions in preventing viral invasion of the brain. To validate the role of mosquito Sni in regulating ROS levels and BBB permeability, we knocked down *Sni* in *A*. *albopictus* (*Aalb Sni*) and *A*. *aegypti* (*Aaeg Sni*) through dsRNA injection (p<0.01; S6I and S6J Fig). H_2_O_2_ assays showed that ROS levels were elevated in *Sni*-knockdown mosquitoes (p<0.05; Fig 6D and 6E). Additionally, injection of TR-Dex dye revealed increased accumulation of fluorescent dye in the brains and eyes of *Sni*-knockdown mosquitoes (p<0.01; Fig 6F–6I), indicating that both the BBB and blood-eye-barrier were compromised. We then infected *A*. *albopictus* mosquitoes with SINV via injection and monitored viral load using RT-qPCR and immunofluorescence (Fig 6J). *Sni*-knockdown mosquitoes showed significantly increased SINV viral loads and higher mortality rates post-infection compared to those of the control group (p<0.05; Fig 6K and 6L), confirming the role of *Sni* in antiviral defense in mosquitoes. Immunofluorescence showed significant viral presence in the brain, midgut, and salivary glands of *Sni*-knockdown mosquitoes (p<0.01; Figs 6M, 6N and S6K–S6O).

In the mosquito-borne virus transmission cycle, viruses enter the mosquito via the blood meal, then infect the mosquito and break through the midgut barrier, spread throughout the body, replicate profusely in the salivary glands, and eventually spread to a new host through mosquito bites [89]. To investigate whether *Sni* impacts viral transmission in mosquitoes, we examined viral loads in various tissues of *Sni*-knockdown *A*. *aegypti* mosquitoes after natural SINV infection via blood-feeding (Fig 6O). Plaque assays indicated increased viral loads in *Sni*-knockdown mosquitoes compared to controls (Fig 6P). Importantly, RT-qPCR and immunofluorescence results showed higher viral loads and infection rates in the heads and guts of *Sni*-knockdown mosquitoes compared to controls (p<0.05; Figs 6Q, 6R and S6P–S6R). Furthermore, Smurf assay revealed that *sni* knockdown led to increased intestinal permeability in mosquitoes, with a higher proportion of “Smurf mosquitoes” than that in the control group (*Luc* dsRNA 3.6% vs *Sni* dsRNA 9.1%; p = 0.0196; S6S and S6T Fig). These results indicate that *Sni* has a conserved antiviral function in mosquitoes, and knocking down *Sni* increases SINV transmission by enabling the virus to break through the intestinal barrier and spread throughout the body of mosquitoes. Taken together, these results suggest that Sni in mosquitoes protects the brain from SINV infection by suppressing ROS and maintaining BBB integrity (Fig 6S).

## Discussion

Brain is the central neural hub that controls biting behavior of mosquitoes, directly influencing the success of viral transmission from mosquito saliva to the host [90]. In this study, we identified a previously unrecognized molecular mechanism through which the Sni protein protects the BBB by regulating ROS levels, thereby preventing arbovirus brain infections in vector insects. As a carbonyl reductase, Sni converts reactive carbonyl compounds into less harmful alcohols, thereby reducing their toxicity. This process helps maintain redox balance and mitigates oxidative damage within the organism [82]. We identified a new *sni*^*EY20992*^ mutant that exhibited significantly elevated ROS levels and increased viral infection. Pharmacological restoration of ROS levels in this mutant effectively restored BBB integrity and alleviated viral infection in the brain. These findings suggest that, unlike traditional antioxidant enzymes such as SOD and Cat, Sni modulates ROS levels through its carbonyl reducing activity, highlighting a unique oxidative stress countermeasure in the antioxidant defense system of vector insects.

The infection hypothesis suggests that chronic viral infections in the CNS could be an underlying cause of Alzheimer disease [74]. Recent studies indicate that exposure to 45 distinct viruses is closely associated with a higher risk of neurodegenerative diseases, with viral encephalitis showing the strongest link to Alzheimer disease [91]. Consistent with the phenotype of *sni*-null mutants [82], our results demonstrate that the *sni*^*EY20992*^ mutant succumbs to oxidative stress and exhibits neurodegenerative signs such as brain vacuolation. Four weeks post-SINV infection, these mutants show markedly reduced locomotor ability, supporting the infection hypothesis. TR-Dex dye assay demonstrated that the loss of *sni* expression led to elevated ROS levels, which compromised the BBB integrity in *Drosophila*, subsequently increasing viral infection of the brain. However, genetic and pharmacological reduction of ROS levels restored BBB integrity and prevented viral infection. In the brains of *sni* mutant flies, the correlation between increased viral infection and neurodegenerative phenotypes suggested that ROS may directly damage neural cells and disrupt the BBB, leading to infection and inflammation, which in turn may trigger neurodegenerative diseases. This insight highlights the role of Sni in protecting neural cells from ROS-induced damage and may have significant implications for preventing and treating neurodegenerative diseases.

Additionally, we observed that increased BBB permeability in *Sni*-depleted flies was associated with disrupted SJ structure, as indicated by the structural markers Dlg1, NrxIV, and Nrg, alongside a reduction in Dlg1 protein expression. This suggests that elevated ROS levels may compromise BBB integrity by destabilizing the SJ structure. Interestingly, we found that the loss of Sni function in glial cells, particularly in SPG cells, was sufficient to increase viral load in the brain, highlighting the critical role of Sni in suppressing ROS production within SPG cells for BBB integrity. Given that increased ROS levels lead to both lipid droplet accumulation and lipid peroxidation in glial cells [55–57], it is worth investigating whether ROS-induced lipid peroxidation and lipid droplet accumulation specifically occur in BBB glial cells and contribute to SJ destabilization, thereby compromising BBB integrity. In mammals, multiple signaling pathways maintain the integrity of the BBB to defend against viral entry into the brain [68,69]. For instance, the receptors Mertk and Axl can cooperate with type I IFN signaling to activate Rac1, stabilizing endothelial tight junctions to tighten the BBB and prevent viral invasion into the CNS [68]. Similarly, IFN-λ signaling enhances BBB integrity by modulating the localization of two key tight junction proteins, ZO-1 and Claudin-5, through a noncanonical STAT1-independent pathway. This process tightens the BBB structure, effectively restricting viral neuroinvasion and subsequent pathogenesis [69]. In *Drosophila*, in addition to essential Moody/protein kinase A signaling for BBB formation and maturation during embryonic and larval stages [92], the miR-285–Yki/Mask double-negative feedback loop has also been found critical for larval BBB development [88]. However, it is not clear how the BBB maintains its integrity and resists viral invasion in the adult brain. Our findings suggest that reinforcing BBB integrity through SJ proteins to protect against viral invasion in the adult brain is a conserved mechanism shared by insects and mammals.

Mosquitoes are the key vectors for numerous arboviruses. Their antiviral immunity not only protects them from fatal virus-induced damage but also influences viral transmission efficiency and arboviral disease spread among humans [22–24,93]. Therefore, understanding the antiviral immune mechanisms in mosquitoes is essential for predicting and controlling arboviral outbreaks. We observed that Sni expression is induced by viral infection in mosquitoes, suggesting that Sni, as an antioxidant factor, may be influenced by upstream antiviral signaling. Our previous study demonstrated that NF-κB and STING-dependent antiviral responses play a critical role in brain defenses against viral infections in *Drosophila* [33], suggesting that further studies should evaluate whether Sni is regulated by NF-κB or related pathways. Additionally, we found that the *sni* gene shows high sequence similarity across *Drosophila*, mosquitoes, and humans. Knockdown of *sni* expression in mosquito brains and human U2OS cells led to increased viral replication, highlighting its evolutionally conserved antiviral function. Studies on the BBB in insects other than *Drosophila* are scarce. It has been shown that the parasite Acanthamoeba can invade locust brains by compromising the integrity of their BBB [94], suggesting that insect BBBs may have a protective function against pathogens similar to that in mammals. Our study validated these findings in the vector mosquitoes. We found that, in wild-type mosquitoes, the TR-Dex dye was effectively blocked from penetrating the brain. In contrast, in *sni*-knockdown mosquitoes, the dye breached the BBB and entered the brain, accompanied by a significant increase in SINV viral load post-infection. These findings provide the first evidence that the BBB in mosquitoes protects against brain viral infections, underscoring its essential function in safeguarding the brain from pathogen invasion. Furthermore, we observed a significant increase in viral load in the brains and intestines of *Sni*-knockdown mosquitoes orally infected with SINV. A Smurf assay also revealed that a higher proportion of these mosquitoes exhibit intestinal barrier disruption and increased permeability. In humans, oxidative stress in the intestine triggers barrier dysfunction, contributing to the onset and progression of various intestinal disorders, including inflammatory bowel disease and colorectal cancer. [95]. High-fat diets can disrupt gut barrier integrity through gut microbiota-derived ROS, compromising barrier function by downregulating tight junction proteins [96]. Similar to mammals, our study demonstrated that Sni protects intestinal barrier integrity in mosquitoes by regulating ROS levels. In *Sni*-deficient mosquitoes, elevated ROS levels may compromise the gut barrier, allowing the virus to breach and disseminate more easily, subsequently leading to infections in the brain and salivary glands and potentially facilitating transmission to the next host. These results suggest that the antioxidant defense system in mosquitoes, which involves Sni, SOD, and Cat, not only protects against ROS-induced tissue damage but also modulates the dissemination of arboviruses within the mosquito, indirectly influencing the viral transmission cycle in nature. Targeting these enzymes could control viral replication in mosquitoes, thereby effectively interrupting the transmission of arboviruses in nature. This discovery not only enhances our understanding of antiviral mechanisms in mosquitoes but also provides new strategies for managing arboviral diseases, which has significant public health implications.

## Materials and methods

### Cells and virus

U2OS, Vero E6 and BHK-21 cells were obtained from ATCC and maintained in Dulbecco’s modified Eagle medium (DMEM, Gibco, 11995065) supplemented with 10% heat-inactivated fetal bovine serum (FBS, Gibco, A3160902), 1% L-glutamine (Gibco, 25030081), and 1% penicillin/streptomycin (Gibco, 15140122) at 37°C in 5% CO_2_ in humidified CO_2_ incubators. Aag-2 cells were a kind gift from Dr. Han Xia (Wuhan Institute of Virology, Chinese Academy of Sciences), and were maintained at 28°C in Schneider’s *Drosophila* medium (Gibco, 21720024) supplemented with 10% FBS (Gibco), 1% L-glutamine (Gibco), and 1% penicillin/streptomycin (Gibco) at 28°C in humidified incubators. C6/36 cells were obtained from ATCC, and were maintained in Leibovitz’s L-15 (Gibco, 11415064) supplemented with 10% FBS (Gibco), 1% L-glutamine (Gibco), and 1% penicillin/streptomycin (Gibco) at 28°C in the absence of CO_2_ in humidified incubators. All cell lines were confirmed negative for mycoplasma contamination. A recombinant Sindbis virus (HRsp) that expresses GFP under control of a subgenomic promoter, was generated by transfecting BHK-21 cells with RNA transcribed in vitro from a full-length cDNA clone as described [97,98]. The virus was propagated in C6/36 cells and viral titers were determined by plaque assay using Vero E6 cells.

### RNAi and virus infection

For RNAi experiments in Aag-2 cells, dsRNAs were synthesized in vitro using the T7 High Efficiency Transcription Kit (Transgene, JT101-02) with gene-specific primers (listed in S3 Table), and purified using an RNA Purification Kit (Transgene, ER101-01) following the manufacturer’s protocol. Aag-2 cells (2×10^5^ cells per well) were seeded in 24-well plates and transfected with 500 ng/well dsRNA using Effectene Transfection Reagent (Qiagen, 301425) following the manufacturer’s protocol. Plates were incubated for 48 hours and then the cells were infected with SINV at a MOI of 1 for 24 hours. For RNAi experiments in U2OS cells, siRNA was synthesized by Tsingke Biotech (listed in S3 Table). U2OS cells (2×10^5^ cells per well) were seeded in 24-well plates and transfected with siRNA using Lipofectamine 2000 (Invitrogen, 11668019) with a final siRNA concentration of 20nM following the manufacturer’s protocol. Plates were incubated for 48 hours and then the cells were infected with SINV at a MOI of 2 for 24 hours. RNA was harvested using Trizol reagent (Invitrogen, 15596018CN) for RT-qPCR analysis.

### Fly husbandry and infections

All fly stocks used in this study are Wolbachia-free and listed in the S2 Table. Flies were reared on standard cornmeal medium (the recipe for 1 Liter food is: cornmeal 50 g, yeast 18.75 g, sucrose 80g, glucose 20 g, agar 5 g, and propionic acid 30 ml) at 25°C on a 12-hour light-dark cycle. All experiments used 4- to 7-day-old adult female flies, unless otherwise stated. A daily heat shock was applied by incubating the flies at 37°C for 45 minutes to activate *HS-Gal4* expression. Mated female flies (4–7 days old) were used for all adult experiments. Flies of the indicated genotypes were inoculated with a 50 nL volume of virus suspension containing 10^6^ pfu/ml of SINVGFP by intrathoracic injection (FemtoJet 4i, Eppendorf) as previously described [33]. For fly infection, tissues were collected at 7 days post infection (dpi) and SINV viral loads were measured by RT-qPCR, western blotting or immunostaining.

### AD4 feeding assay

For the AD4 feeding assay, N-acetylcysteine amide (AD4; Macklin, N872752) was added to standard fly food at a concentration of 40 μg/ml. The control group received food with an equivalent amount of PBS. Flies were introduced to these diets at the hatching stage. After eclosion, they were transferred to a fresh vial with the same diet every 3 days until dissection.

### Climbing assay

To assess the locomotor ability of *sni*-depleted flies, a climbing assay was performed as described [99,100]. Briefly, groups of female flies of the indicated genotypes were placed in 10 cm high empty plastic vials. The vial was gently tapped on the table to knock all the flies to the bottom, and they were left standing for 8 seconds. The number of flies that climbed to the top or bottom half of the vial was recorded and scored. Flies that scaled the top half of the vial walls were scored as 1, while those remaining in the bottom half were scored as 0. Throughout the testing process, the observer conducting the test was blinded to the genotypes of the flies. The climbing ability of the flies was monitored in three replicates for each experiment.

### Mosquito rearing

*A*. *aegypti* (Liverpool strain) and *A*. *albopictus* (Foshan strain) mosquitoes were kindly provided by Dr. Chun-Hong Chen (National Health Research Institutes, Taiwan) and Dr. Shaohui Liang (School of Basic Medical Sciences, Wenzhou Medical University). Adult mosquitoes were maintained in a temperature and humidity-controlled environment at 28°C and 75 ± 5% relative humidity, with a regulated 12-hour light-dark cycle. Larvae were reared at 28°C and fed a balanced diet consisting of a 1:1 ratio of yeast powder and goose liver powder. Male and female adult mosquitoes were housed together in a cage and provided with a 10% sucrose solution. For all experiments, female mosquitoes aged 1–3 days were used.

### Mosquito gene silencing and infection

Female mosquitoes aged 1–3 days were anesthetized with CO_2_ and injected intrathoracically with approximately 0.3 μg of dsRNA per mosquito using the FemtoJet 4i injection system (Eppendorf). After three days, the mosquitoes were injected with 50 nL of viral suspension containing 10^5^ pfu/ml of SINVGFP. For oral infection, mosquitoes were fed a 1:1 mixture of defibrinated sheep blood and virus, containing 5×10^6^ pfu/ml of SINVGFP, for 2 hours using a glass mosquito feeder. Gene silencing efficiency was assessed via RT-qPCR. Survival assays were performed on groups of at least 15 mosquitoes, maintained at 28°C post-infection, with mortality rates recorded every day.

### Plaque assay

For the plaque assay, each individual mosquito orally infected with SINV was collected at 8 dpi and homogenized in 300 μL of DMEM. The homogenate was centrifuged at 12,000 g for 5 minutes, and 4 μL of the filtered supernatant was used to infect Vero E6 cells cultured in 24-well plates. The plates were incubated with DMEM containing 10% FBS (Gibco, A3160902), 1% L-glutamine (Gibco, 25030081), 1% penicillin/streptomycin (Gibco, 15140122) and 1% methylcellulose (Sigma, M0512) for 3 days at 37°C in 5% CO_2_ in humidified CO_2_ incubators. Plaques were fixed with 4% formaldehyde, stained with 1% crystal violet (Sigma, C6158), and counted under a microscope. At least three biological replicates were performed.

### TR-Dex penetration assay

TR-Dex penetration assay was performed as previously described [62,84]. Approximately 300 nL of a 25 mg/mL 10 kDa Texas red-conjugated dextran solution (TR-Dex; Invitrogen, D1863) was injected into the body cavity of *Drosophila* or mosquitoes. Following injection, insects were allowed to recover for 24 hours before fixation in 4% formaldehyde for 80 minutes. Samples were then washed in 0.1% PBST prior to brain dissection, which was conducted and examined on the same day. Brains were mounted in Vectashield Mounting Media (Vector Laboratories) and imaged using a Leica SP8 confocal laser scanning microscope under the same settings with an HC PL APO CS2 20x/0.75 DRY objective lens. For each set of experiments, images were acquired as single image sections captured at a resolution of 1024 × 1024 pixels (0.758 μm × 0.758 μm), with a pixel dwell time of 0.4 microseconds and frame averaging at 3% laser power. The pinhole size was set to 169.8 micrometers. Imaging setting were kept consistent across all experiments to ensure comparable results.

To assess blood-eye barrier integrity in mosquitoes, a similar protocol was followed. Mosquitoes were injected with 300 nL of TR-Dex solution (25 mg/mL), and after 24 hours, mosquito heads were excised and imaged using a Zeiss Axioscope 5 microscope under the same settings with an EC Plan-Neofluar 10x/0.3 M27 objective and a Hamamatsu digital camera.

### Hydrogen Peroxide assay for determination of ROS activity

Insect bodies or heads were homogenized using a Beyotime Hydrogen Peroxide Assay Kit (Beyotime Biotechnology, S0038) following the manufacturer’s instructions. After homogenization, samples were centrifuged at 12,000 g for 5 minutes. The supernatant was collected and analyzed using the assay kit. Fluorescence intensity was measured at an emission wavelength of 560 nm using the Cytation3 Multifunctional Enzyme Labeler (BioTek).

### DHE assay

To assess oxidative stress of *Drosophila* brains, a dihydroethidium (DHE) assay was performed as previously described [101,102]. Brains were dissected in Schneider’s *Drosophila* medium (Gibco, 21720024) supplemented with 10% FBS (Gibco, A3160902). The dissected brains were incubated in 30 μM DHE (Invitrogen, D1168) for 5 minutes, washed three times with PBS, and then fixed in 4% formaldehyde for 8 minutes. After fixation, brains were mounted in Vectashield Mounting Media (Vector Laboratories) and single image sections were captured using a Zeiss Axioscope 5 microscope under the same settings with an EC Plan-Neofluar 10x/0.3 M27 objective and a Hamamatsu digital camera.

### RT-qPCR

Total RNA was extracted from *Drosophila* (6 whole bodies, 15 heads and 15 guts per genotype) or cells using Trizol reagent (Invitrogen, 15596018CN) according to the manufacturer’s protocol. For mosquito tissues, RNA was extracted using the RNA simple Total RNA Kit (Tiangen Biotech, DP419). First-strand cDNA was synthesized using the HiScript III RT SuperMix Reverse Transcription Kit (Vazyme, R323-01). Real-time PCR was performed in triplicate for each sample using SYBR Green (Vazyme, Q311-02) on a QuantStudio 6 System (Thermo Fisher Scientific) with gene-specific primers listed in S3 Table. The relative abundance of gene transcripts was normalized to *GAPDH* (*Homo sapiens*), *rp49* (*Drosophila*), *Rps7* (*A*. *albopictus* AALF016123) or *Rps17* (*A*. *aegypti* AAEL025999) as endogenous controls using the 2^-ΔΔCT^ method.

### Immunostaining and fluorescence microscopy

Intestines, salivary glands, and brains were dissected in PBS and fixed in 4% formaldehyde for 30 minutes. Samples were washed three times in PBS with 0.3% Triton X-100, for 10 minutes each wash with shaking. They were then blocked in Immunol Staining Blocking Buffer (Beyotime Biotechnology, P0102) for 45 minutes and incubated with primary antibodies diluted in blocking buffer at 4°C overnight with shaking. The primary antibodies used were: mouse anti-discs large at 1:50 (DSHB, 4F3), mouse anti-GFP at 1:500 (Roche, 11814460001), rat anti-Elav at 1:50 (DSHB, 7E8A10), mouse anti-Repo at 1:50 (DSHB, 8D12), and chicken anti-GFP at 1:2000 dilution (Abcam, ab13970). After washing three times in wash buffer, tissues were incubated with secondary antibodies and DAPI for 2 hours at room temperature with shaking, followed by the same washing steps. The secondary antibodies (Alexa 488 and Alexa 568, Invitrogen) were used at a 1:1000 dilution, and nuclei were stained with DAPI (Sigma, D9542) at 1 μg/ml. Brain immunostaining with anti-Dlg1 in larvae, as well as NrxIV-GFP and Nrg-GFP reporter staining in adult flies, was performed as previously described [63,84,88]. For NrxIV-GFP and Nrg-GFP reporter staining, brains from flies of the specified genotypes were dissected in PBS, fixed in 4% formaldehyde for 30 minutes, and washed three times with PBS. Samples were then mounted in Vectashield Mounting Media (Vector Laboratories) and imaged at room temperature (25°C) using a Leica SP8 confocal laser scanning microscope under the same settings with HC PL APO CS2 20x/0.75 DRY and HC PL APO CS2 63x/1.40 OIL objectives.

### Primary neural cell culture and immunological fluorescence assay

Primary neural cells derived from *Drosophila* larval brains were cultured following previously established protocols [103,104]. Briefly, late third instar larvae were collected, rinsed twice in 70% ethanol, and washed three times with sterile PBS. Larval brains were dissected in Schneider’s *Drosophila* medium (Gibco, 21720024) with 1% penicillin/streptomycin (Gibco), and imaginal discs were carefully removed to ensure purity of neural cells in culture. After dissection, brains were washed three times with Rinaldini solution (8 mg/ml NaCl, 0.2 mg/ml KCl, 0.05 mg/ml NaH₂PO₄·H₂O, 1 mg/ml NaHCO₃, and 1 mg/ml glucose in ddH₂O) and incubated with 0.5 mg/ml collagenase type IV (Gibco) for 1 hour at 37°C. Following collagenase treatment, brains were washed three times in Schneider’s *Drosophila* medium, dissociated, and plated onto 96-well plates for incubation at 25°C in a humidified cell culture incubator.

After 24 hours of incubation, cells were infected with SINVGFP at a MOI of 1. Cell suspensions were transferred onto Teflon-coated slides 24 hours post-infection, fixed in 4% formaldehyde for 15 minutes, and washed three times with 0.1% Triton X-100. Samples were blocked with Immunol Staining Blocking Buffer (Beyotime Biotechnology, P0102) for 45 minutes, then incubated overnight at 4°C with primary antibodies in blocking buffer. Primary antibodies included rat anti-Elav (1:50, DSHB, 7E8A10), mouse anti-Repo (1:50, DSHB, 8D12), and chicken anti-GFP (1:2000, Abcam, ab13970). After washing three times with wash buffer, samples were incubated for 2 hours at room temperature with secondary antibodies (Alexa 488 and Alexa 568, Invitrogen, 1:1000) and DAPI (Sigma, D9542, 1 μg/ml) for nuclear staining. Samples were mounted in Vectashield Mounting Media (Vector Laboratories) and imaged using a Leica SP8 confocal laser scanning microscope under the same settings with HC PL APO CS2 63x/1.40 OIL objective.

### Smurfs assay

The Smurfs assay of mosquitoes were performed following previously established protocol [105]. In brief, female mosquitoes were injected with *Luc* dsRNA or *Sni* dsRNA and allowed to recover for three days. They were then starved for 24 hours and subsequently fed a solution of 10% glucose mixed with 2% (w/v) FD&C blue dye #1 for an additional 24 hours. Mosquitoes showing a significant presence of dye throughout their bodies, including legs and abdomen, were classified as “Smurf”, and the percentage of these mosquitoes was recorded.

### Immunoblotting

For immunoblotting, 15 *Drosophila* intestines, 15 adult heads, 6 whole bodies, and 20 L3 larval brains were collected for each experiment and lysed with RIPA buffer (50 mM Tris-HCl, pH 8.0, 150 mM NaCl, 2 mM MgCl_2_, 1.5% NP-40, 0.1% SDS, and 0.5% sodium deoxycholate) supplemented with protease inhibitor cocktail (Sigma, P7626). The samples were homogenized with zirconium beads and then placed on ice for 20 minutes. Lysates were centrifuged at 12,000 g for 15 minutes to collect the supernatant. Protein concentrations were determined using a BCA kit (Beyotime Biotechnology, P0011). Total protein from each sample was mixed with loading buffer (Beyotime Biotechnology, P0015L) and boiled for 10 minutes. The proteins were resolved on SDS–PAGE and transferred onto polyvinylidene difluoride (PVDF) membranes. After blocking with 5% non-fat milk in TBST for 1 hour and washing twice (10 minutes each), the PVDF membranes were incubated with primary antibodies with shaking overnight at 4°C and washed three times with TBST (10 minutes each). The PVDF membranes were incubated with shaking at room temperature for 2 hours with horseradish peroxidase-labeled secondary antibody and washed three times with TBST (10 minutes each). Primary antibodies used were mouse anti-GFP (1:2000; Roche, 11814460001), rabbit anti-β-actin (1:5000; ABclonal, ARC5115-01), and mouse anti-discs large (1:500; DSHB, 4F1). Secondary antibodies were horseradish peroxidase-conjugated Peroxidase AffiniPure Goat Anti-Mouse IgG (H+L) (1:5000; Jackson ImmunoResearch, 115-035-003) and Peroxidase AffiniPure Goat Anti-Rabbit IgG (H+L) (1:5000; Jackson ImmunoResearch, 111-035-003). The results shown are representative of at least three independent experiments.

### Quantification and Statistical Analysis

#### SINVGFP fluorescence quantification

For each set of experiments comparing SINVGFP fluorescence intensity, control and experimental groups of the flies and mosquitoes were dissected, fixed, and immunostained under the same conditions. Confocal images of single image sections were captured using a Leica SP8 confocal laser scanning microscope under the same settings. SINVGFP fluorescence area were quantified by calculating the area of the SINVGFP in the raw confocal images using the Area function in ImageJ. Briefly: Open image: File → Open. Split Channels: image→ color →Split Channels. Keep the channel to be calculated and delete other channels. Set scale to make sure the unit of length is Pixel: Analyze →Set scale →click to remove scale. Set measurements. Analyze →Set Measurements. Tick the boxes marked “Area,” “Integrated Density,” and “limit to threshold.” Then click on the “OK” button. Select the ROI using any of the drawing/selection tools. Tick the boxes marked “measure” to calculate the area intensity for both the DAPI and SINVGFP channels. The ratio of SINVGFP/DAPI (Fluorescent Area), as shown in Figs 2F, 2J, 6N, S2C, S2I, S2L, S6M and S6O, was calculated by dividing the area of SINVGFP by the area of DAPI.

#### DHE fluorescence quantification

For each set of experiments comparing DHE fluorescence intensity, flies from the control and the experimental groups were dissected and subsequently incubated in 30 μM DHE, fixed, and immune-stained under the same conditions. Samples were mounted in Vectashield Mounting Media (Vector Laboratories) and DHE staining images were captured using Zeiss Axioscope 5 microscope under the same settings. The whole brain area of each raw DHE-stained images was analyzed in ImageJ to determine mean pixel intensity. The fold change in DHE fluorescence intensity, as shown in Figs 3C, S3C and S3G, was calculated by measuring the average intensity for each group, comparing it to the average intensity of the control group, and calculating the fold change relative to the control.

#### TR-Dex quantification

For each experiment comparing brain TR-Dex intensity, control and experimental groups of the flies and mosquitoes were dissected, fixed, and immunostained under the same conditions. Samples were mounted in Vectashield Mounting Media (Vector Laboratories) and confocal images of single image sections were captured using a Leica SP8 confocal laser scanning microscope under the same settings. Brains were imaged on the same day of fixation to minimize dextran diffusion. Average intensity was measured of the raw confocal images from the central plane of the brain or eye region using ImageJ. Fold change in TR-Dex intensity, as illustrated in Figs 4B, 4E, 4G, 5F, 6G, 6I, S4B, S4D and S6H, was calculated by measuring the average intensity for each group, comparing it to the average intensity of the control group, and determining the fold change accordingly.

#### Quantification of SJ width and the proportion of disrupted SJs

For each experiment comparing SJ width and the proportion of disrupted SJs, flies from both control and experimental groups were dissected, fixed, and immunestained under the same conditions. Samples were mounted in Vectashield Mounting Media (Vector Laboratories) and confocal images of single image sections were captured using a Leica SP8 confocal laser scanning microscope under the same settings. The widths of NrxIV-GFP and Nrg-GFP-labeled SJ belts, as depicted in Figs 5K, 5N and S5F, were measured by randomly selecting three different SJ areas in the raw confocal images of each brain. The width of these SJs was measured using the length measurement tool in Leica LAS X Microscope Imaging Software, and the average of the three measurements was recorded as the SJ width for that brain. Multiple SJ belts from at least 20 brains were analyzed to ensure objectivity and minimize potential bias.

For the quantification of the ratio of brain with disrupted SJ belts (Fig 5B, 5D, 5J and 5M), a brain (NrxIV-GFP and Nrg-GFP) or hemisphere (Dlg1) with two or more disruptions was classified as SJ belt disrupted brain. The proportion of SJ belt disrupted brains was then calculated by dividing the number of brains with disrupted SJ belts by the total number of brains analyzed.

#### Band Gray Value statistics of western blotting

The average gray values of both the target protein and the internal reference protein were measured using ImageJ (Figs 1H, 1K, 2D, 2H, 3F, 3I, 3L, 5G, S2E, S2F and S5D). For each group, the ratio of the target protein’s gray value to that of the internal reference protein was calculated. To normalize these results across experimental groups, the ratio in the control group was used as the baseline reference value.

#### Statistical analysis

Data were analyzed by unpaired two-tailed Student’s t-test or one-way ANOVA, unless otherwise noted. The Log-Rank test was used for survival analysis. The nonparametric Mann-Whitney test was used for Figs 6K, 6P, 6Q, S6D and S6F, and the Chi-squared test was used for Figs 6R and S6R. Thresholds of statistical significance were set as follows: *p < 0.05, **p < 0.01, ***p < 0.001, ****p < 0.0001, and ns for non-significance (p > 0.05). Error bars represent the SEM of three independent experiments. Images were processed using Adobe Photoshop 2024 and Adobe Illustrator 2024 for image merging and resizing. Fluorescence intensity in visual fields was calculated using ImageJ software. Statistical analysis was performed using GraphPad Prism 8, Microsoft Excel, and IBM SPSS Statistics 23. All source data, including uncropped western blot images and raw data for all figures and tables, are provided in S1 File.

## Supporting information

S1 Fig Candidate genetic screening identified Sni as an antiviral factor. **Related to Fig 1. (**A and B) RT-qPCR validation of knockdown efficiency in cells. RT-qPCR analysis of indicated gene mRNA expression in human U2OS cells (A) and mosquito Aag-2 cells (B) treated with siRNA/dsRNA targeting specific genes for 48 hours, followed by infection with SINV for 24 hours. (C and D) RT-qPCR validation of knockdown efficiency in *sni* RNAi flies. (E) The schematic representation of *sniffer* and *Trxr1* loci. (F and G) *sni* and *Trxr1* mRNA expression levels in *sni* mutant flies. (H) Representative HE staining images of heads from 25-day-old control (*w*^*1118*^) or the *sni* mutant (*sni*^*EY20992*^) flies. Arrowheads indicate vacuoles in the head. (I) Quantification of vacuole number in heads of control (*w*^*1118*^) or *sni* mutant (*sni*^*EY20992*^) flies. The numbers of quantified *Drosophila*: 10 (*w*^*1118*^) and 10 (*sni*^*EY20992*^). (J) Percent survival of control (*w*^*1118*^ and *sni*^*EY20992/+*^) or *sni* mutant (*sni*^*EY20992*^) flies under hyperoxia. The numbers of quantified *Drosophila*: 80 (*w*^*1118*^), 72 (*sni*^*EY20992/+*^), and 72 (*sni*^*EY20992*^). (K) Climbing assay measuring locomotor ability in *w*^*1118*^ and *sni*^*EY20992*^ flies with or without SINV infection. The numbers of quantified *Drosophila*:14d: 86 (*w*^*1118*^ UN), 75 (*sni*^*EY20992*^ UN), 87 (*w*^*1118*^ SINV), and 67 (*sni*^*EY20992*^ SINV); 21d: 95 (*w*^*1118*^ UN), 75 (*sni*^*EY20992*^ UN), 86 (*w*^*1118*^ SINV), and 77 (*sni*^*EY20992*^ SINV); and 28d: 81 (*w*^*1118*^ UN), 72 (*sni*^*EY20992*^ UN), 43 (*w*^*1118*^ SINV), and 46 (*sni*^*EY20992*^ SINV). Data represent mean ± SEM. Scale bars represent 50 μm (H). Statistical analysis was performed using two-tailed unpaired Student’s t-test (A–D, F, G and I), One-way ANOVA (K) and Log-Rank test (J). At least three independent experiments were performed.
(TIF)

S2 Fig Sni restricts SINV infection in the brain and gut of adult *Drosophila*. **Related to Fig 2.** (A) RT-qPCR analysis of SINV viral load in heads of SINV-infected *sni* RNAi (*tub>sni*^*RNAi#1*^) or control (*tub>+*) flies at 7 dpi. (B) Representative immunofluorescence images of heads stained for SINVGFP in SINV-infected control (*tub>+*) or *sni* RNAi (*tub>sni*^*RNAi#1*^) flies at 7 dpi (virus, green; nuclei, blue). (C) Quantification of SINVGFP fluorescence area in (B). The numbers of quantified *Drosophila* heads: 13 (*tub >+*) and 13 (*tub >sni*^*RNAi#1*^). (D) Representative immunofluorescence images of *sni* mutant fly heads stained for SINVGFP with Elav or Repo at 7 dpi (virus, green; Elav or Repo, red). Arrowheads indicate the co-localization of SINVGFP with Elav or Repo. (E and F) Western blot analysis of SINV viral load in 15 pooled guts from *sni* RNAi (E) and *sni* mutant (F) and control flies at 7 dpi. (G) RT-qPCR analysis of SINV viral load in 15 pooled guts from *sni* RNAi or control flies at 7 dpi. (H and I) Representative immunofluorescence images and quantification of SINV infected guts in *sni* RNAi (*HS>sni*^*RNAi#2*^) and control (*HS>+*) flies at 7 dpi. (J) RT-qPCR analysis of SINV viral load in 15 pooled guts from *sni* mutant or control flies at 7 dpi. (K and L) Representative immunofluorescence images and quantification of SINV infected guts from *sni* mutant (*sni*^*EY20992*^) or control (*w*^*1118*^) flies at 7 dpi. Data represent mean ± SEM. In (B, H and K), dotted line indicates the edge of the *Drosophila* brains and guts. Scale bars represent 50 μm (B, D, H and K). Statistical analysis was performed using two-tailed unpaired Student’s t-test (A, C, G, I and L) and One-way ANOVA (J). At least three independent experiments were performed.
(TIF)

S3 Fig Sni restricts viral infection by modulating ROS levels in the brain. **Related to Fig 3.** (A) Schematic timeline of AD4 treatment and DHE assay shown in (B). (B) Representative immunofluorescence images of L3 larval brains stained with DHE for control (*w*^*1118*^) or *sni* mutant (*sni*^*EY20992*^) flies fed with 40 μg/ml AD4 or PBS (DHE, red). (C and D) Quantification of DHE intensity (C) and brain area (D) in (B). *w*^*1118*^ flies fed with PBS were used as control. The numbers of quantified brains from left to right are 16, 17, 16, and 16. (E) ROS activity measured by H_2_O_2_ assay in 25 pooled heads of control (*tub>+*) or *sni* RNAi (*tub>sni*^*RNAi#1*^) flies. (F) Representative immunofluorescence images of adult brains stained with DHE for control (*tub>+*) and *sni* RNAi (*tub>sni*^*RNAi#1*^) flies. (G) Quantification of DHE intensity in (F). The numbers of quantified brains from left to right are 32 and 29. (H) Western blot analysis of Sni-Flag expression in *sni* overexpression (*Act>sni*) or control (*Act>+*) fly heads. β-actin was used as a loading control. Data represent mean ± SEM. Scale bars represent 50 μm (B and F). Statistical analysis was performed using two-tailed unpaired Student’s t-test (E and G), One-way ANOVA (C and D). At least three independent experiments were performed.
(TIF)

S4 Fig Depletion of *sni* led to ROS-induced disruption of BBB integrity in the brain. **Related to Fig 4.** (A) Representative immunofluorescence images of *Drosophila* heads stained with TR-Dex for control (*tub>+*) or *sni* RNAi (*tub>sni*^*RNAi#1*^) flies (TR-Dex, red). (B) Quantification of TR-Dex intensity in (A). *tub>+* flies were used as controls. The numbers of quantified *Drosophila* heads: 32 (*tub>+*) and 24 (*tub>sni*^*RNAi#1*^). (C) Representative immunofluorescence images of *Drosophila* heads stained with TR-Dex for control (*HS>+*) or *Sod2* RNAi (*HS >Sod2*^*RNAi*^) flies (TR-Dex, red). (D) Quantification of TR-Dex intensity in (C). *HS>+* flies were used as controls. The numbers of quantified *Drosophila* heads: 22 (*HS>+*) and 23 (*HS>Sod2*^*RNAi*^). (E) RT-qPCR analysis of SINV viral load in the heads of SINV-infected *Sod2* RNAi (*HS>Sod2*^*RNAi*^) and control (*HS>+*) at 7 dpi. Data represent mean ± SEM. Scale bars represent 50 μm (A and C). In (A and C), dotted line indicates the edge of the *Drosophila* head. Statistical analysis was performed using two-tailed unpaired Student’s t-test (B, D and E). At least three independent experiments were performed.
(TIF)

S5 Fig The depletion of *sni* led to disrupted SJ integrity in the BBB. **Related to Fig 5.** (A) RT-qPCR analysis of SINV viral load in 15 pooled heads from SINV infected BBB-disrupted (*moody>moody*^*RNAi*^ and *moody>NrxIV*^*RNAi*^) or control (*moody>+*) flies at 7 dpi. (B) Representative immunofluorescence images of *Drosophila* heads stained with Dlg1 in *moody>GFP*.*nls* and *PG>GFP*.*nls* flies (GFP, green; nuclei, blue; Dlg1, red). (C) RT-qPCR analysis of SINV viral load in 15 pooled heads from SINV infected flies with *sni* knocked down in specific brain cell types. (D) Western blot analysis of Dlg1 expression in 20 pooled L3 larval brains of *sni* mutant (*sni*^*EY20992*^) or control (*w*^*1118*^) flies. β-actin was used as a loading control. (E) Representative immunofluorescence images of adult brains stained with Nrg-GFP in control (*HS>+*) or *sni* RNAi (*HS>sni*^*RNAi#2*^) flies. (F) Quantification of Nrg-GFP width shown in (E). *HS>+* flies were used as controls. The numbers of quantified *Drosophila* heads:20 (*HS>+*) and 27(*HS>sni*^*RNAi#2*^), Data represent mean ± SEM. Scale bars represent 50 μm (B) and 10 μm (E). Statistical analysis was performed using two-tailed unpaired Student’s t-test (A, C and F). At least three independent experiments were performed.
(TIF)

S6 Fig The mosquito Sni homolog protects BBB integrity against SINV infection by suppressing ROS. **Related to Fig 6.** (A) RT-qPCR analysis of *Sni* mRNA level in SINV infected mosquitoes and C6/36 cells at 1 dpi. (B) RT-qPCR analysis of *Sod2* mRNA level in control (*Luc* dsRNA) or *Sod2* knockdown *A*. *aegypti* mosquitoes 3 days post-dsRNA injection. Each dot represents 6 pooled mosquitoes. (C) ROS activity in *Sod2* knockdown *A*. *aegypti* mosquitoes and control (*Luc* dsRNA). ROS activity was measured by H_2_O_2_ assay. Each dot represents 6 pooled mosquitoes. (D) RT-qPCR analysis of SINV viral load in the heads of SINV infected control (*Luc* dsRNA) or *Sod2* knockdown (*Sod2* dsRNA) mosquitoes at 3 dpi. Each dot represents an individual mosquito. The numbers of quantified mosquitoes: 44 (*Luc* dsRNA) and 42 (*Sod2* dsRNA). (E) RT-qPCR analysis of *Dlg1* and *NrxIV* knockdown efficiency in *A*. *aegypti* mosquitoes treated with dsRNA targeting specific genes for 3 days. Each dot represents 6 pooled mosquitoes. (F) RT-qPCR analysis of SINV viral load in the heads of SINV infected control (*Luc* dsRNA), *Dlg1* and *NrxIV* knockdown *A*. *aegypti* mosquitoes at 3 dpi. Each dot represents an individual mosquito. The numbers of quantified mosquitoes: 23 (*Luc* dsRNA), 30 (*Dlg1* dsRNA) and 22 (*NrxIV* dsRNA). (G and H) Representative immunofluorescence images (G) and quantification (H) of eyes with TR-Dex staining for control (*Luc* dsRNA) and *Dlg1* or *NrxIV* knockdown mosquitoes. The yellow line represents the edge of the mosquito eye. The numbers of quantified *A*. *aegypti* eyes: 33 (*Luc* dsRNA), 42 (*Dlg1* dsRNA) and 29 (*NrxIV* dsRNA). (I and J) RT-qPCR analysis of *Aaeg Sni* (I) or *Aalb Sni* (J) mRNA expression in control (*Luc* dsRNA) or *sni* knockdown mosquitoes 3 days post-dsRNA injection. Each dot represents 6 pooled mosquitoes. (K) Experimental scheme of SINV infection via injection in *A*. *albopictus*. (L and M) Representative images (L) and quantification (M) of SINVGFP in salivary glands of SINV infected (by injection) control (*Luc* dsRNA) or *Sni* knockdown mosquitoes at 3 dpi (virus, green; nuclei, blue). The numbers of quantified mosquitoes: 10 (*Luc* dsRNA) and 13 (*Sni* dsRNA). (N and O) Representative images (N) and quantification (O) of SINVGFP in midguts of SINV infected (by injection) control (*Luc* dsRNA) or *Sni* knockdown mosquitoes at 3 dpi (virus, green; nuclei, blue). The numbers of quantified mosquitoes: 27 (*Luc* dsRNA) and 24 (*Sni* dsRNA). (P) Experimental scheme of SINV oral infection in *A*. *aegypti*. (Q and R) Representative images (Q) and quantification (R) of SINVGFP in midguts of SINV infected (by oral infection) control (*Luc* dsRNA) or *Sni* knockdown mosquitoes at 8 dpi (virus, green; nuclei, blue) The numbers of quantified mosquitoes: 41 (*Luc* dsRNA) and 39 (*Sni* dsRNA). (S) Representative image of a “Smurf mosquito” showing blue dye permeation throughout the body (right). In non-Smurf controls, the blue dye remains restricted to the digestive tract (left). (T) The percent of “Smurf mosquito” in groups of control (*Luc* dsRNA) and *Sni* knockdown mosquitoes. The numbers of quantified mosquitoes: 120 (*Luc* dsRNA) and 108 (*Sni* dsRNA). Data represent mean ± SEM. The dotted line represents the edge of the mosquito midgut (N and Q) and salivary glands (L). Scale bars represent 50 μm (L, N and Q) and 100 μm (G). Statistical analysis was performed using two-tailed unpaired Student’s t-test (A–C, E, H–J, M, O and T); non-parametric Mann-Whitney test (D and F) and Chi-squared test (R). At least three independent experiments were performed.
(TIF)

S1 Table Results of *Drosophila* screening. (XLSX)

S2 Table *Drosophila* strains used in this study. (XLSX)

S3 Table Primers and siRNA used in this study. (XLSX)

S1 File Source data files including uncropped western blot images and raw data for all figures and tables. (ZIP)
