# Supplementary figures and images for: Sniffer restricts arboviral brain infections by regulating ROS levels and protecting blood-brain barrier integrity in Drosophila and mosquitoes
Source: PLoS Pathog. 2024 Dec 16;20(12):e1012797. doi: 10.1371/journal.ppat.1012797 (PMC11684763; doi:10.1371/journal.ppat.1012797)

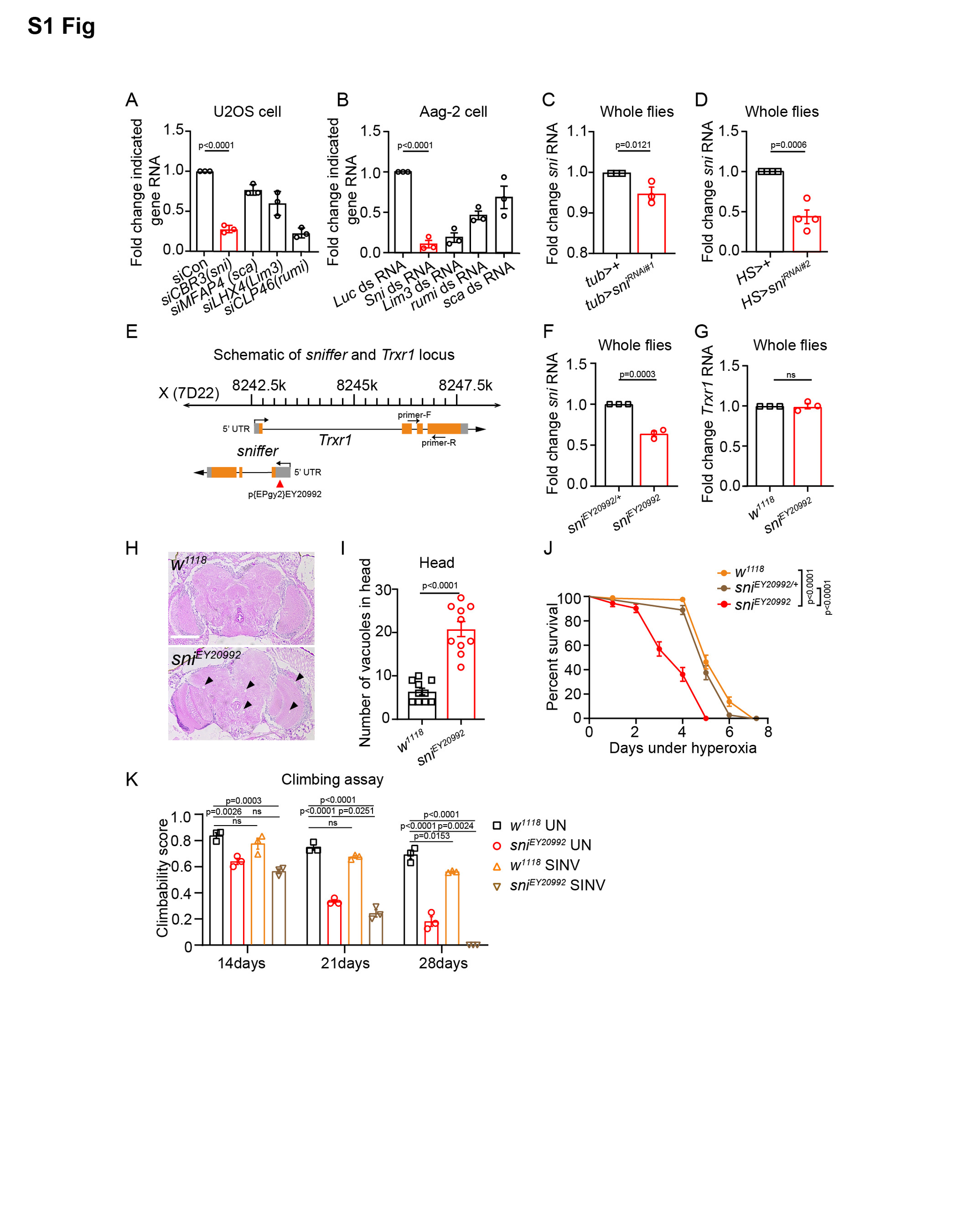

Supplement: S1 Fig — Related to Fig 1. (A and B) RT-qPCR validation of knockdown efficiency in cells. RT-qPCR analysis of indicated gene mRNA expression in human U2OS cells (A) and mosquito Aag-2 cells (B) treated with siRNA/dsRNA targeting specific genes for 48 hours, followed by infection with SINV for 24 hours. (C and D) RT-qPCR validation of knockdown efficiency in sni RNAi flies. (E) The schematic representation of sniffer and Trxr1 loci. (F and G) sni and Trxr1 mRNA expression levels in sni mutant flies. (H) Representative HE staining images of heads from 25-day-old control (w1118) or the sni mutant (sniEY20992) flies. Arrowheads indicate vacuoles in the head. (I) Quantification of vacuole number in heads of control (w1118) or sni mutant (sniEY20992) flies. The numbers of quantified Drosophila: 10 (w1118) and 10 (sniEY20992). (J) Percent survival of control (w1118 and sniEY20992/+) or sni mutant (sniEY20992) flies under hyperoxia. The numbers of quantified Drosophila: 80 (w1118), 72 (sniEY20992/+), and 72 (sniEY20992). (K) Climbing assay measuring locomotor ability in w1118 and sniEY20992 flies with or without SINV infection. The numbers of quantified Drosophila:14d: 86 (w1118 UN), 75 (sniEY20992 UN), 87 (w1118 SINV), and 67 (sniEY20992 SINV); 21d: 95 (w1118 UN), 75 (sniEY20992 UN), 86 (w1118 SINV), and 77 (sniEY20992 SINV); and 28d: 81 (w1118 UN), 72 (sniEY20992 UN), 43 (w1118 SINV), and 46 (sniEY20992 SINV). Data represent mean ± SEM. Scale bars represent 50 μm (H). Statistical analysis was performed using two-tailed unpaired Student’s t-test (A–D, F, G and I), One-way ANOVA (K) and Log-Rank test (J). At least three independent experiments were performed. (TIF) [file ppat.1012797.s001.tif]

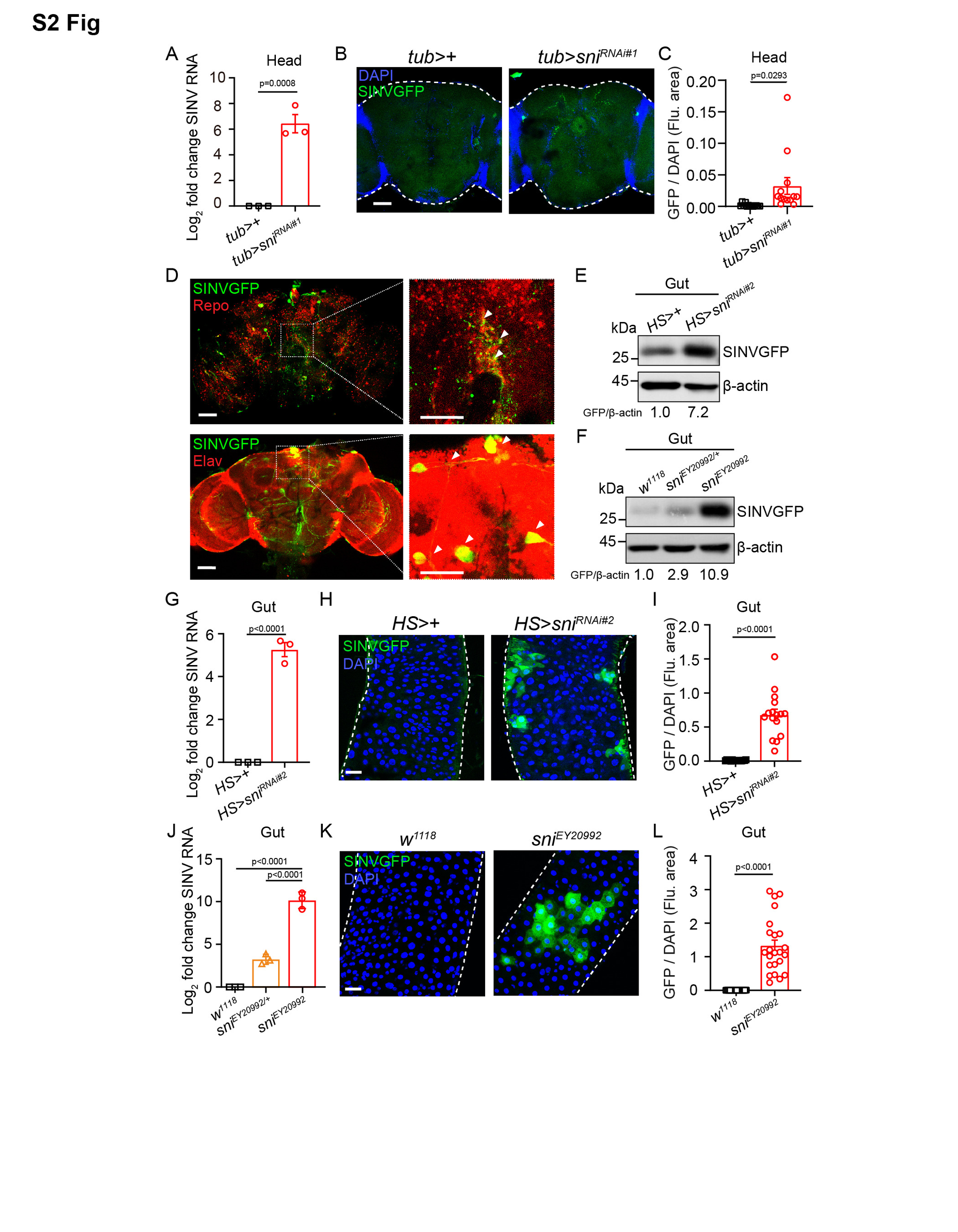

Supplement: S2 Fig — Related to Fig 2. (A) RT-qPCR analysis of SINV viral load in heads of SINV-infected sni RNAi (tub>sniRNAi#1) or control (tub>+) flies at 7 dpi. (B) Representative immunofluorescence images of heads stained for SINVGFP in SINV-infected control (tub>+) or sni RNAi (tub>sniRNAi#1) flies at 7 dpi (virus, green; nuclei, blue). (C) Quantification of SINVGFP fluorescence area in (B). The numbers of quantified Drosophila heads: 13 (tub >+) and 13 (tub >sniRNAi#1). (D) Representative immunofluorescence images of sni mutant fly heads stained for SINVGFP with Elav or Repo at 7 dpi (virus, green; Elav or Repo, red). Arrowheads indicate the co-localization of SINVGFP with Elav or Repo. (E and F) Western blot analysis of SINV viral load in 15 pooled guts from sni RNAi (E) and sni mutant (F) and control flies at 7 dpi. (G) RT-qPCR analysis of SINV viral load in 15 pooled guts from sni RNAi or control flies at 7 dpi. (H and I) Representative immunofluorescence images and quantification of SINV infected guts in sni RNAi (HS>sniRNAi#2) and control (HS>+) flies at 7 dpi. (J) RT-qPCR analysis of SINV viral load in 15 pooled guts from sni mutant or control flies at 7 dpi. (K and L) Representative immunofluorescence images and quantification of SINV infected guts from sni mutant (sniEY20992) or control (w1118) flies at 7 dpi. Data represent mean ± SEM. In (B, H and K), dotted line indicates the edge of the Drosophila brains and guts. Scale bars represent 50 μm (B, D, H and K). Statistical analysis was performed using two-tailed unpaired Student’s t-test (A, C, G, I and L) and One-way ANOVA (J). At least three independent experiments were performed. (TIF) [file ppat.1012797.s002.tif]

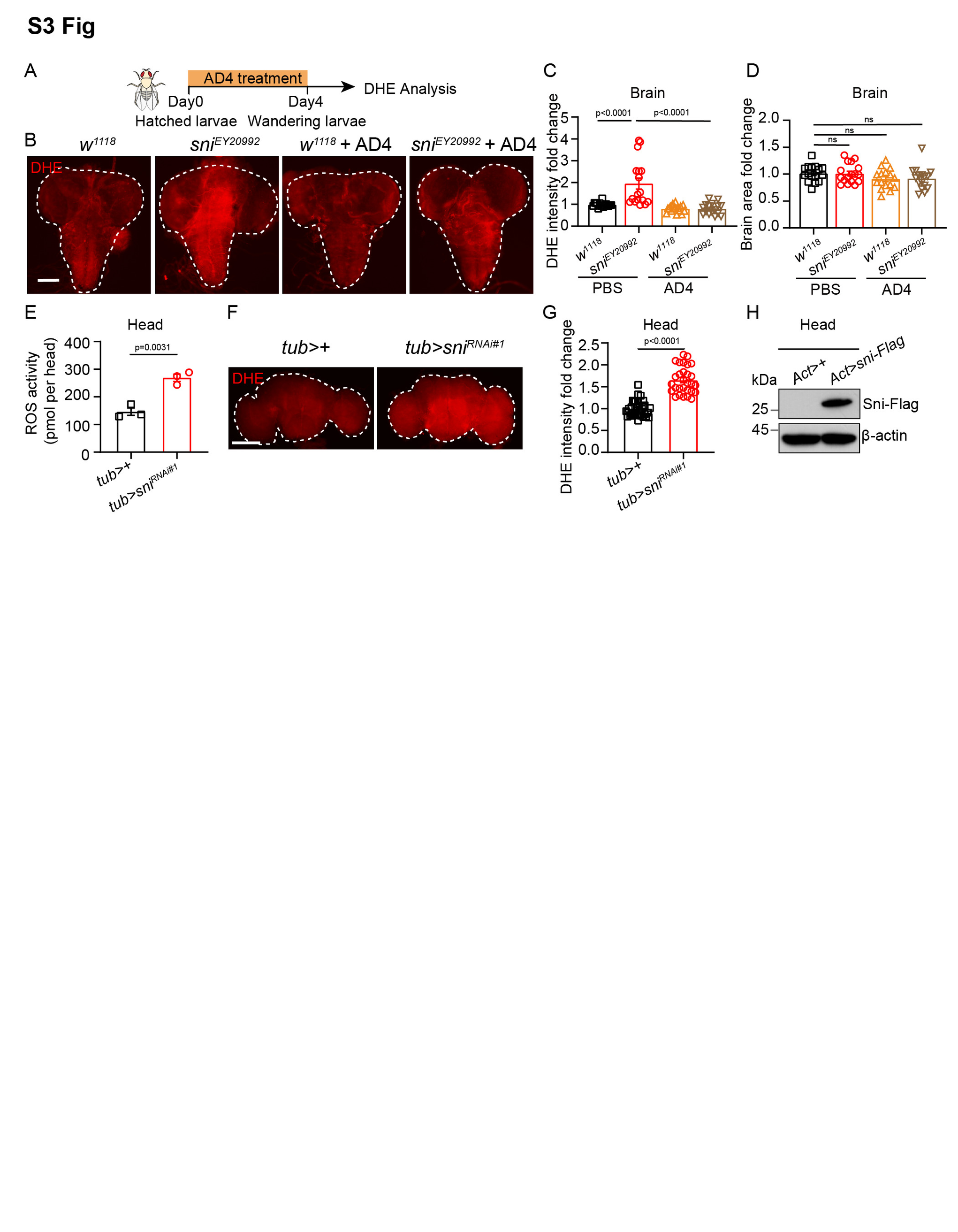

Supplement: S3 Fig — Related to Fig 3. (A) Schematic timeline of AD4 treatment and DHE assay shown in (B). (B) Representative immunofluorescence images of L3 larval brains stained with DHE for control (w1118) or sni mutant (sniEY20992) flies fed with 40 μg/ml AD4 or PBS (DHE, red). (C and D) Quantification of DHE intensity (C) and brain area (D) in (B). w1118 flies fed with PBS were used as control. The numbers of quantified brains from left to right are 16, 17, 16, and 16. (E) ROS activity measured by H2O2 assay in 25 pooled heads of control (tub>+) or sni RNAi (tub>sniRNAi#1) flies. (F) Representative immunofluorescence images of adult brains stained with DHE for control (tub>+) and sni RNAi (tub>sniRNAi#1) flies. (G) Quantification of DHE intensity in (F). The numbers of quantified brains from left to right are 32 and 29. (H) Western blot analysis of Sni-Flag expression in sni overexpression (Act>sni) or control (Act>+) fly heads. β-actin was used as a loading control. Data represent mean ± SEM. Scale bars represent 50 μm (B and F). Statistical analysis was performed using two-tailed unpaired Student’s t-test (E and G), One-way ANOVA (C and D). At least three independent experiments were performed. (TIF) [file ppat.1012797.s003.tif]

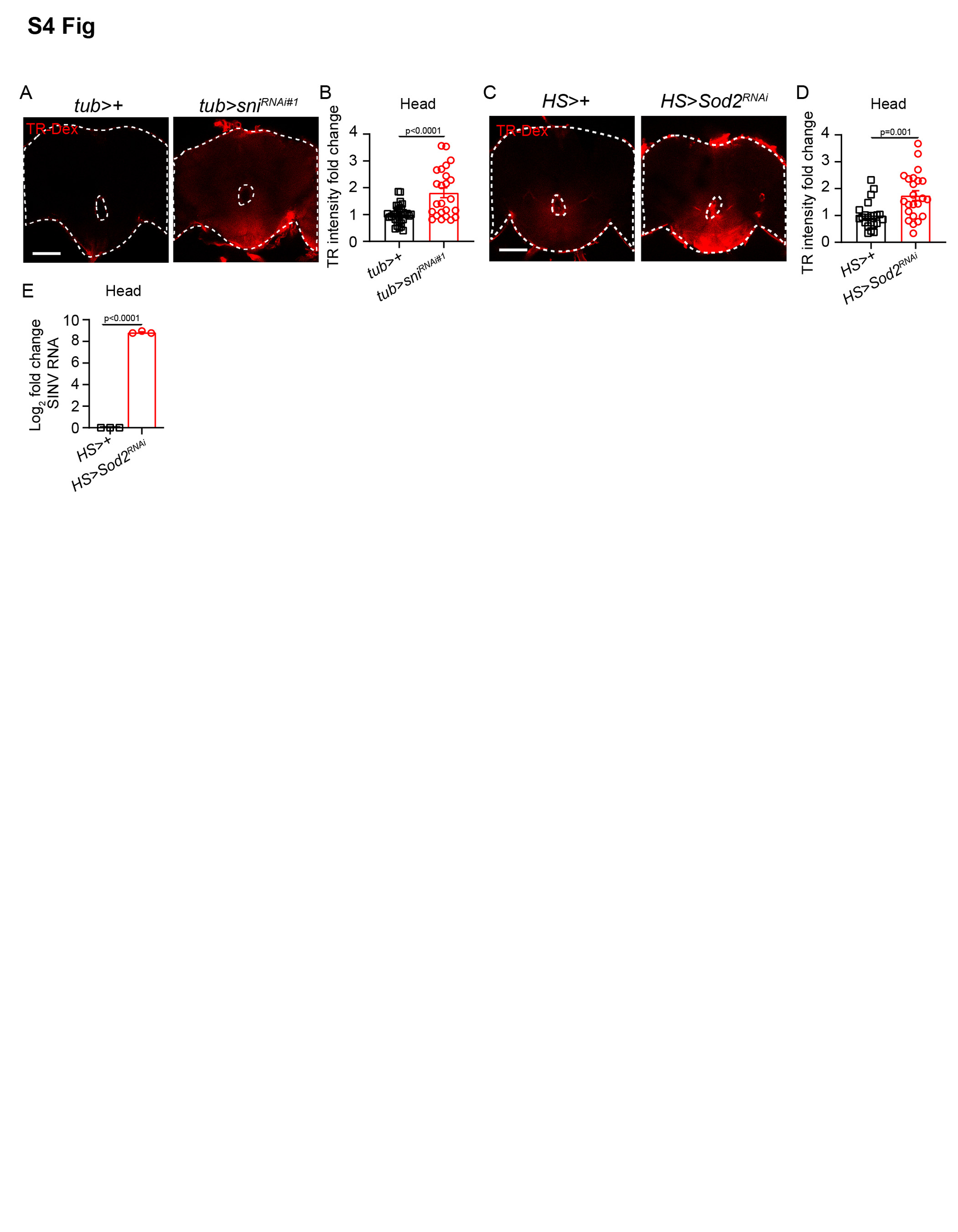

Supplement: S4 Fig — Related to Fig 4. (A) Representative immunofluorescence images of Drosophila heads stained with TR-Dex for control (tub>+) or sni RNAi (tub>sniRNAi#1) flies (TR-Dex, red). (B) Quantification of TR-Dex intensity in (A). tub>+ flies were used as controls. The numbers of quantified Drosophila heads: 32 (tub>+) and 24 (tub>sniRNAi#1). (C) Representative immunofluorescence images of Drosophila heads stained with TR-Dex for control (HS>+) or Sod2 RNAi (HS >Sod2RNAi) flies (TR-Dex, red). (D) Quantification of TR-Dex intensity in (C). HS>+ flies were used as controls. The numbers of quantified Drosophila heads: 22 (HS>+) and 23 (HS>Sod2RNAi). (E) RT-qPCR analysis of SINV viral load in the heads of SINV-infected Sod2 RNAi (HS>Sod2RNAi) and control (HS>+) at 7 dpi. Data represent mean ± SEM. Scale bars represent 50 μm (A and C). In (A and C), dotted line indicates the edge of the Drosophila head. Statistical analysis was performed using two-tailed unpaired Student’s t-test (B, D and E). At least three independent experiments were performed. (TIF) [file ppat.1012797.s004.tif]

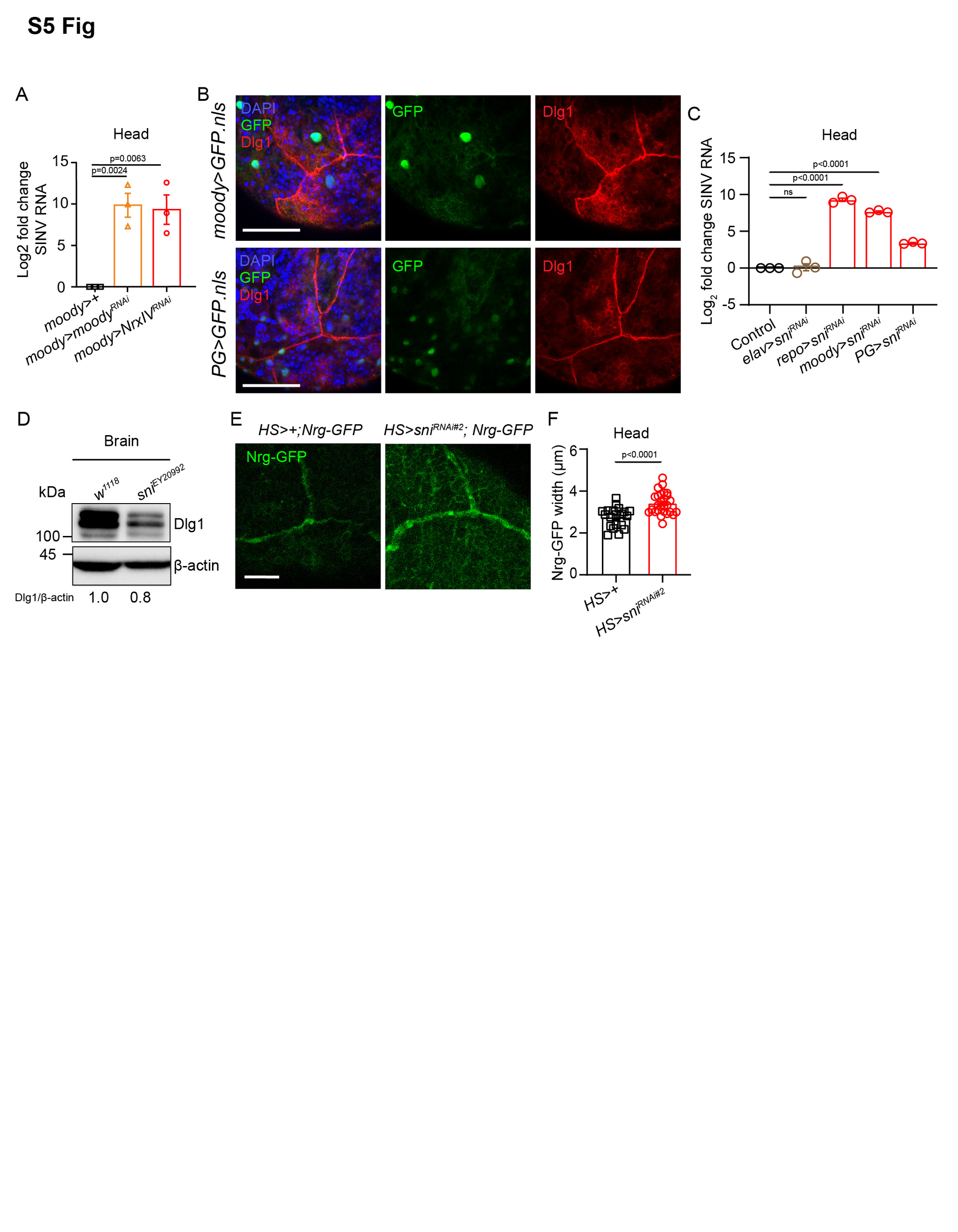

Supplement: S5 Fig — Related to Fig 5. (A) RT-qPCR analysis of SINV viral load in 15 pooled heads from SINV infected BBB-disrupted (moody>moodyRNAi and moody>NrxIVRNAi) or control (moody>+) flies at 7 dpi. (B) Representative immunofluorescence images of Drosophila heads stained with Dlg1 in moody>GFP.nls and PG>GFP.nls flies (GFP, green; nuclei, blue; Dlg1, red). (C) RT-qPCR analysis of SINV viral load in 15 pooled heads from SINV infected flies with sni knocked down in specific brain cell types. (D) Western blot analysis of Dlg1 expression in 20 pooled L3 larval brains of sni mutant (sniEY20992) or control (w1118) flies. β-actin was used as a loading control. (E) Representative immunofluorescence images of adult brains stained with Nrg-GFP in control (HS>+) or sni RNAi (HS>sniRNAi#2) flies. (F) Quantification of Nrg-GFP width shown in (E). HS>+ flies were used as controls. The numbers of quantified Drosophila heads:20 (HS>+) and 27(HS>sniRNAi#2), Data represent mean ± SEM. Scale bars represent 50 μm (B) and 10 μm (E). Statistical analysis was performed using two-tailed unpaired Student’s t-test (A, C and F). At least three independent experiments were performed. (TIF) [file ppat.1012797.s005.tif]

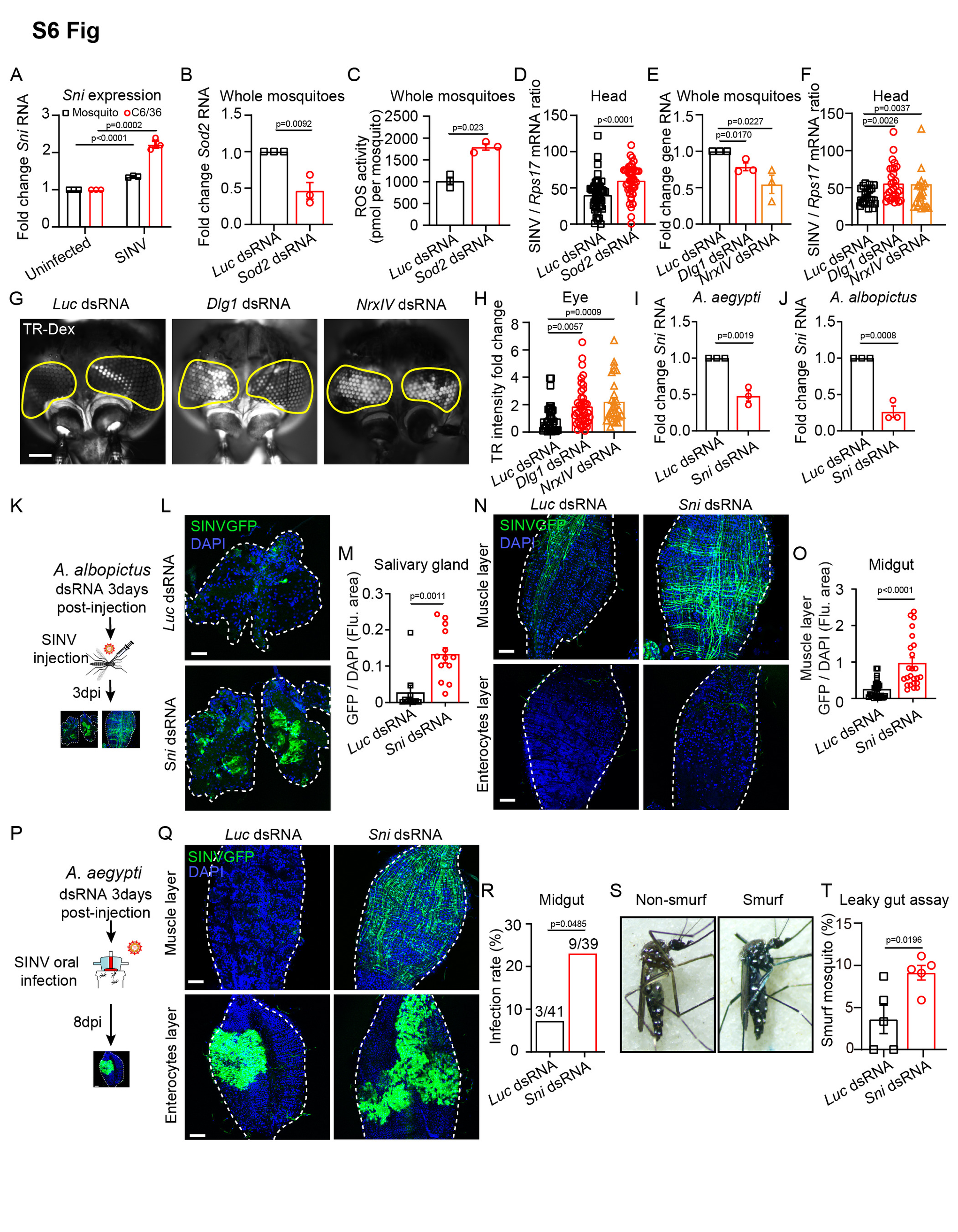

Supplement: S6 Fig — Related to Fig 6. (A) RT-qPCR analysis of Sni mRNA level in SINV infected mosquitoes and C6/36 cells at 1 dpi. (B) RT-qPCR analysis of Sod2 mRNA level in control (Luc dsRNA) or Sod2 knockdown A. aegypti mosquitoes 3 days post-dsRNA injection. Each dot represents 6 pooled mosquitoes. (C) ROS activity in Sod2 knockdown A. aegypti mosquitoes and control (Luc dsRNA). ROS activity was measured by H2O2 assay. Each dot represents 6 pooled mosquitoes. (D) RT-qPCR analysis of SINV viral load in the heads of SINV infected control (Luc dsRNA) or Sod2 knockdown (Sod2 dsRNA) mosquitoes at 3 dpi. Each dot represents an individual mosquito. The numbers of quantified mosquitoes: 44 (Luc dsRNA) and 42 (Sod2 dsRNA). (E) RT-qPCR analysis of Dlg1 and NrxIV knockdown efficiency in A. aegypti mosquitoes treated with dsRNA targeting specific genes for 3 days. Each dot represents 6 pooled mosquitoes. (F) RT-qPCR analysis of SINV viral load in the heads of SINV infected control (Luc dsRNA), Dlg1 and NrxIV knockdown A. aegypti mosquitoes at 3 dpi. Each dot represents an individual mosquito. The numbers of quantified mosquitoes: 23 (Luc dsRNA), 30 (Dlg1 dsRNA) and 22 (NrxIV dsRNA). (G and H) Representative immunofluorescence images (G) and quantification (H) of eyes with TR-Dex staining for control (Luc dsRNA) and Dlg1 or NrxIV knockdown mosquitoes. The yellow line represents the edge of the mosquito eye. The numbers of quantified A. aegypti eyes: 33 (Luc dsRNA), 42 (Dlg1 dsRNA) and 29 (NrxIV dsRNA). (I and J) RT-qPCR analysis of Aaeg Sni (I) or Aalb Sni (J) mRNA expression in control (Luc dsRNA) or sni knockdown mosquitoes 3 days post-dsRNA injection. Each dot represents 6 pooled mosquitoes. (K) Experimental scheme of SINV infection via injection in A. albopictus. (L and M) Representative images (L) and quantification (M) of SINVGFP in salivary glands of SINV infected (by injection) control (Luc dsRNA) or Sni knockdown mosquitoes at 3 dpi (virus, green; nuclei, blue). The numbers of quantifi [file ppat.1012797.s006.tif]
