# Supplementary material for: Sniffer restricts arboviral brain infections by regulating ROS levels and protecting blood-brain barrier integrity in Drosophila and mosquitoes
Source: PLoS Pathog. 2024 Dec 16;20(12):e1012797. doi: 10.1371/journal.ppat.1012797 (PMC11684763; doi:10.1371/journal.ppat.1012797)

Fig1H β-actin

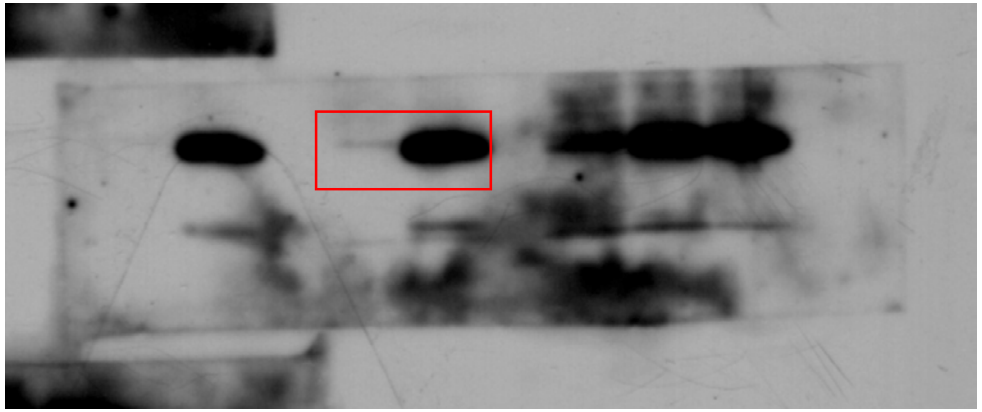

Fig1H GFP

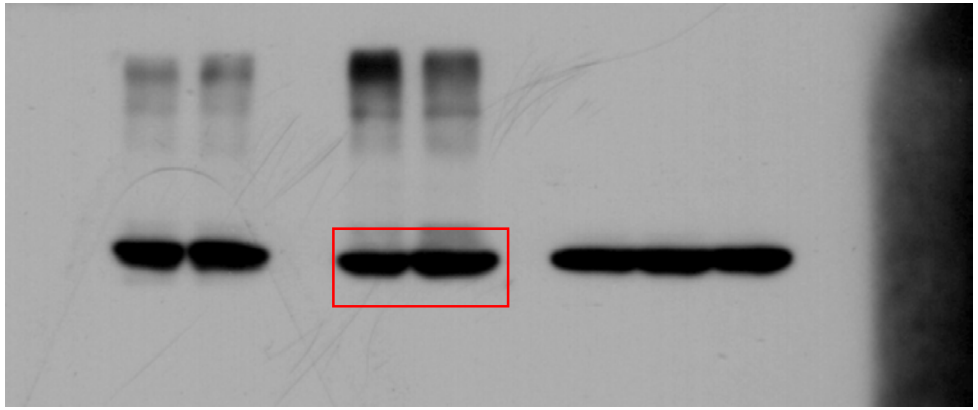

Fig1H Membrane

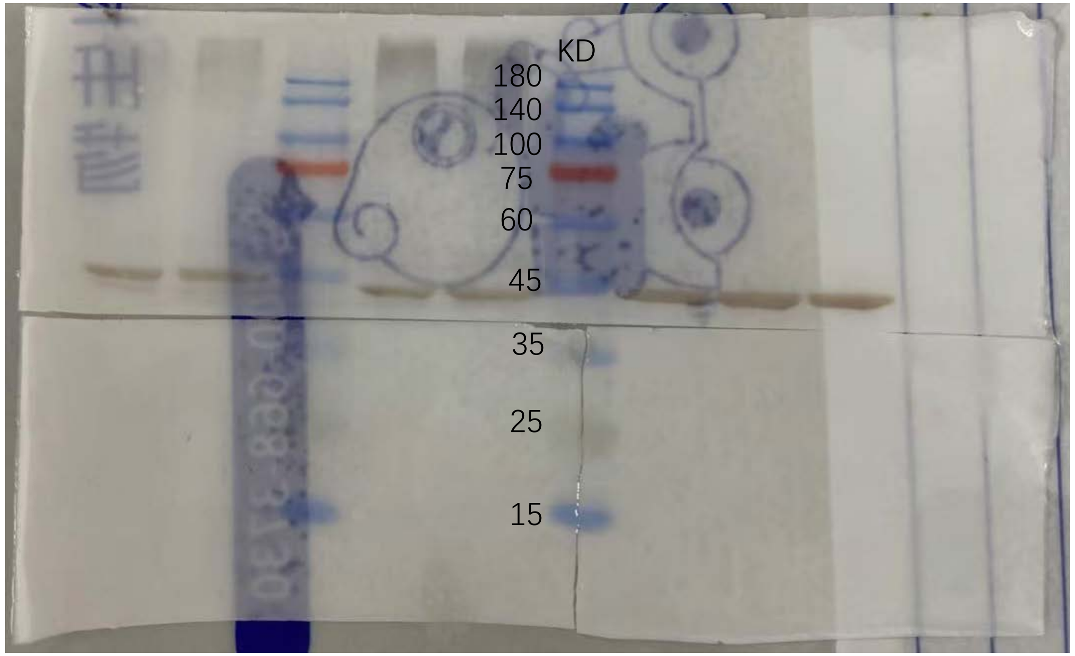

Fig1K β-actin

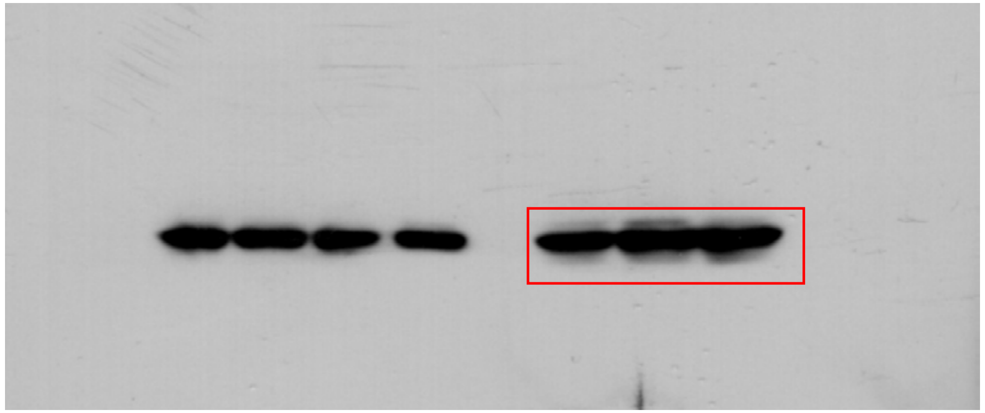

Fig1K GFP

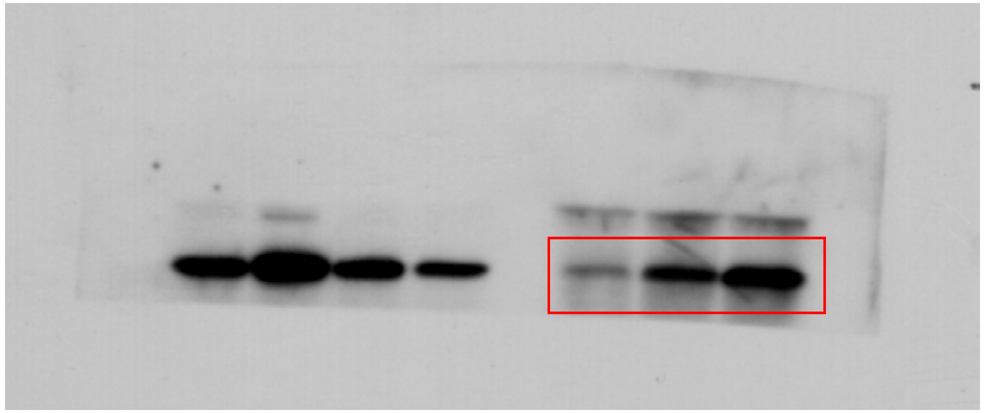

Fig1K Membrane

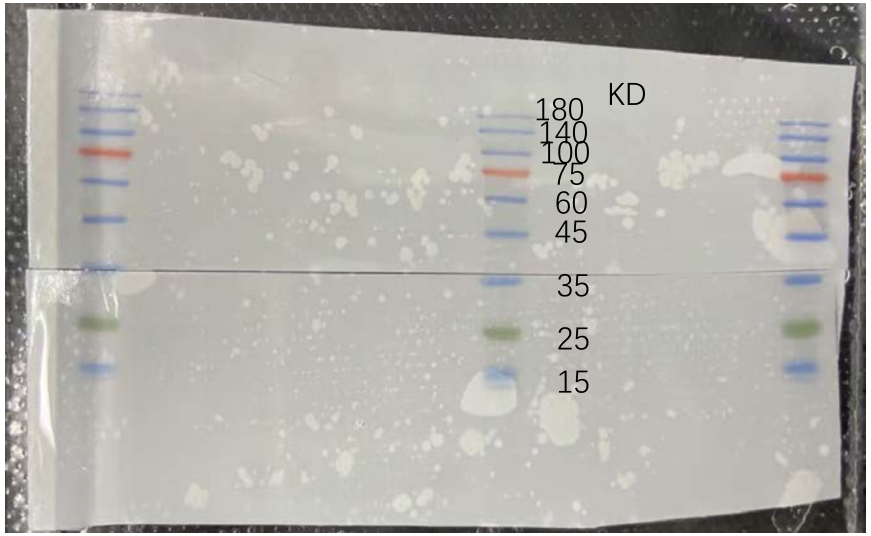

Fig2D β-actin

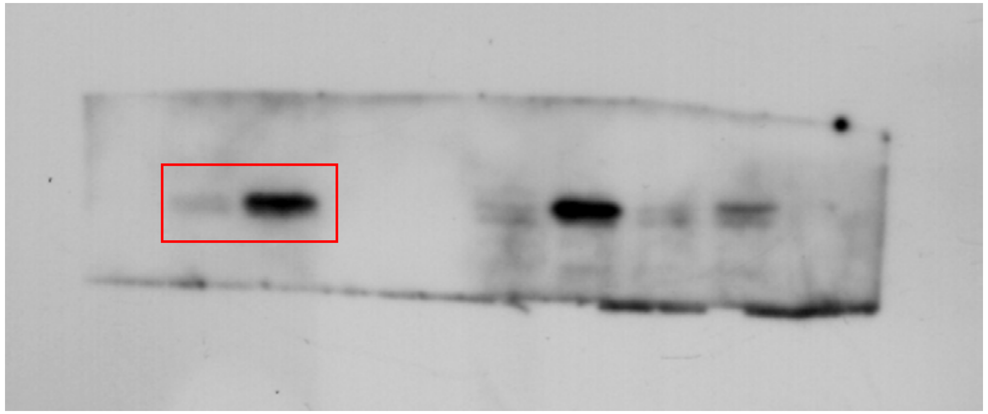

Fig2D GFP

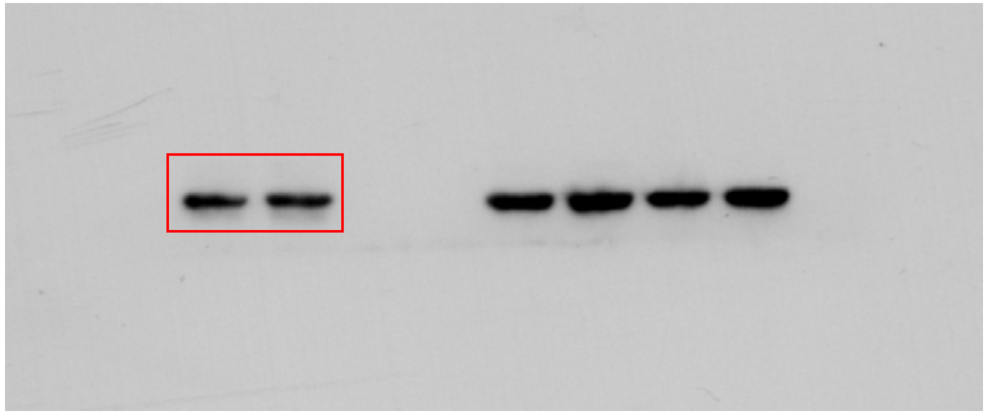

Fig2D Membrane

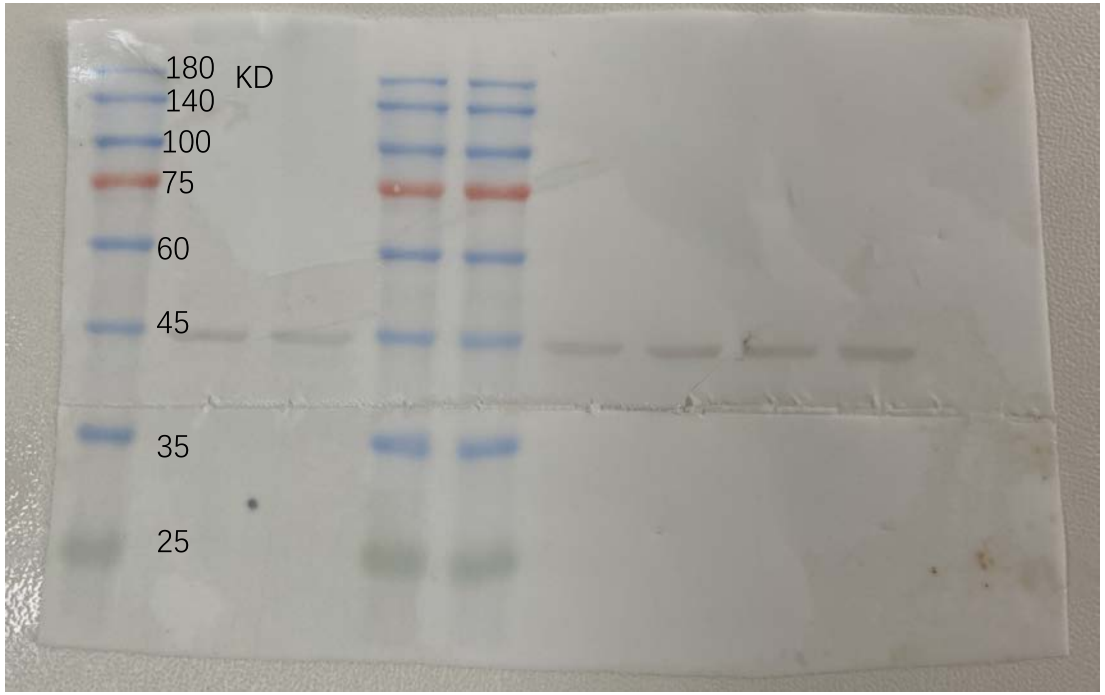

Fig2H β-actin

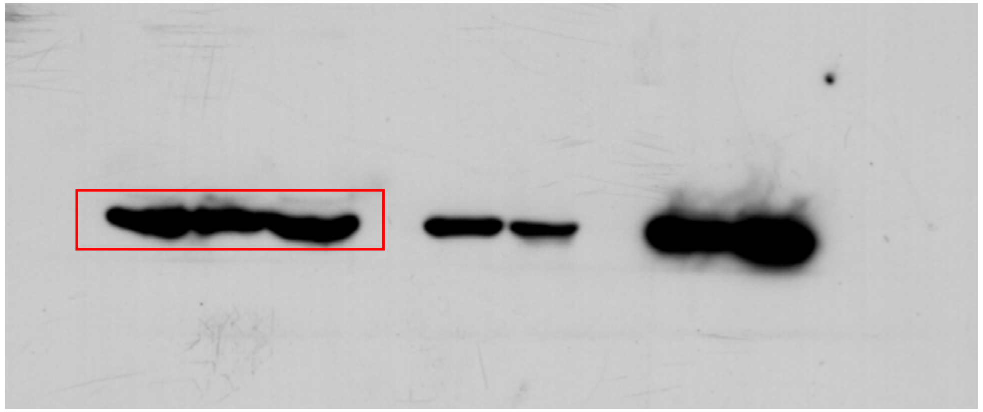

Fig2H GFP

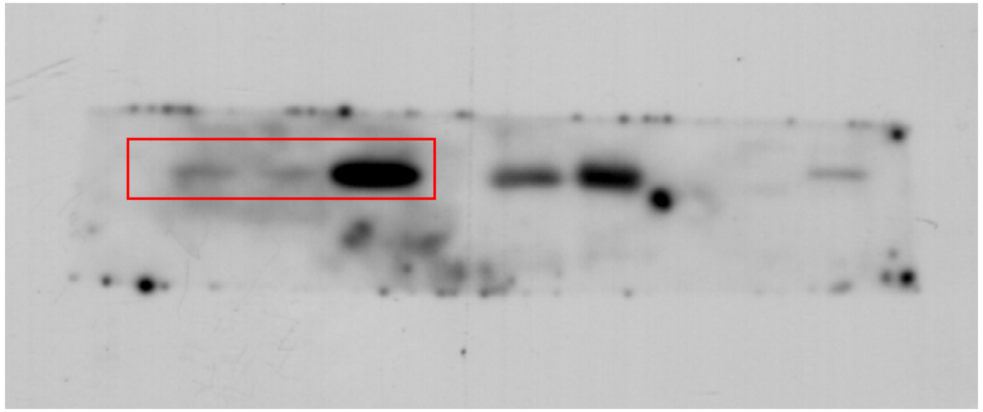

Fig2H Membrane

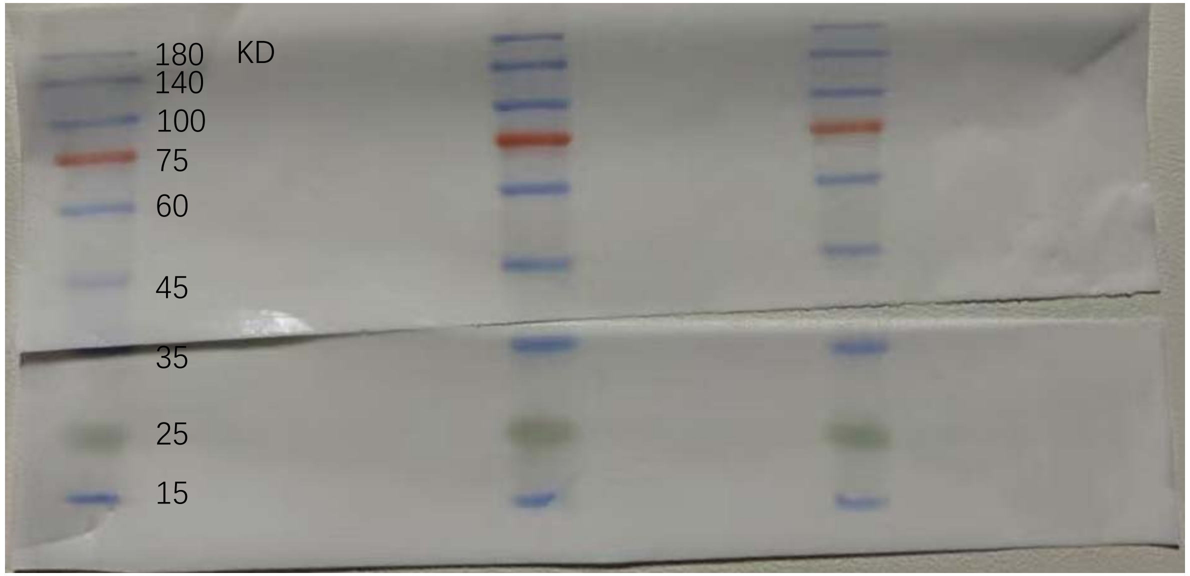

Fig3F β-actin

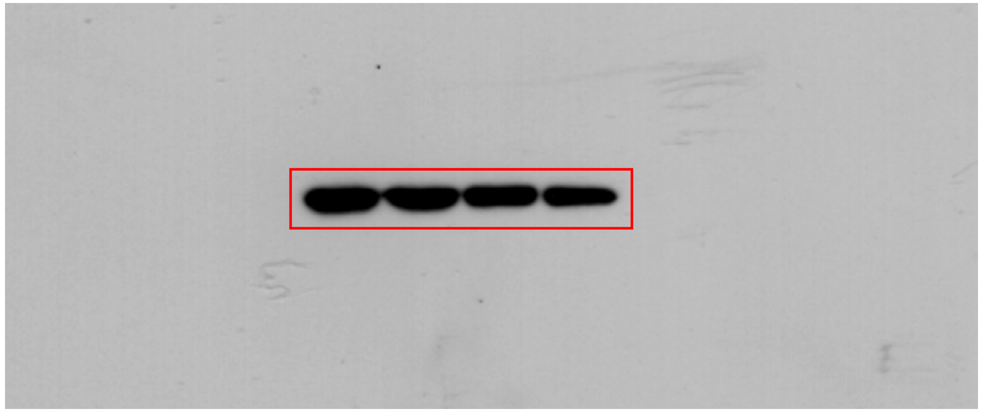

Fig3F GFP

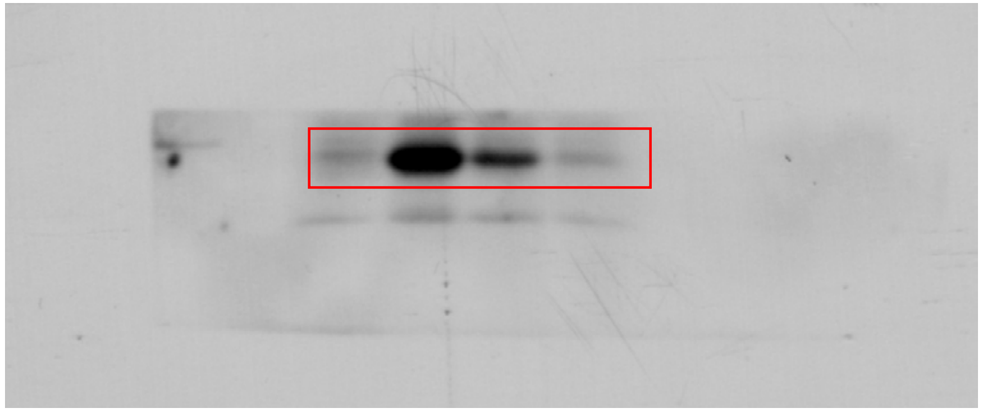

Fig3F Membrane

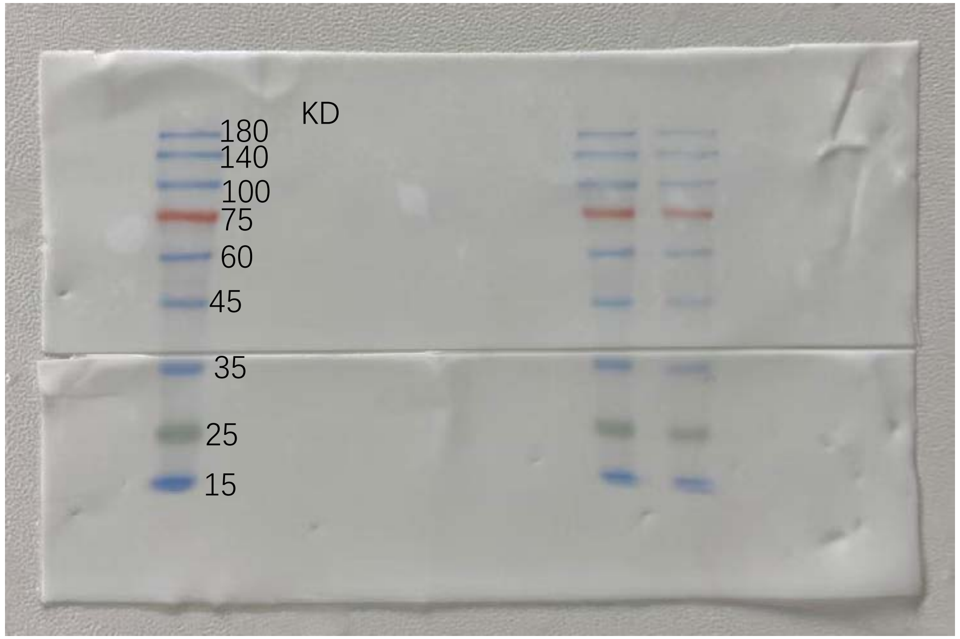

Fig3I β-actin

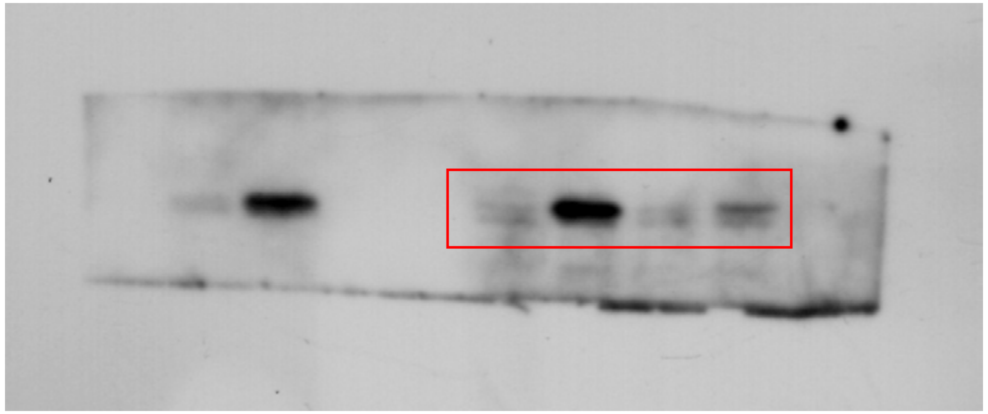

Fig3I GFP

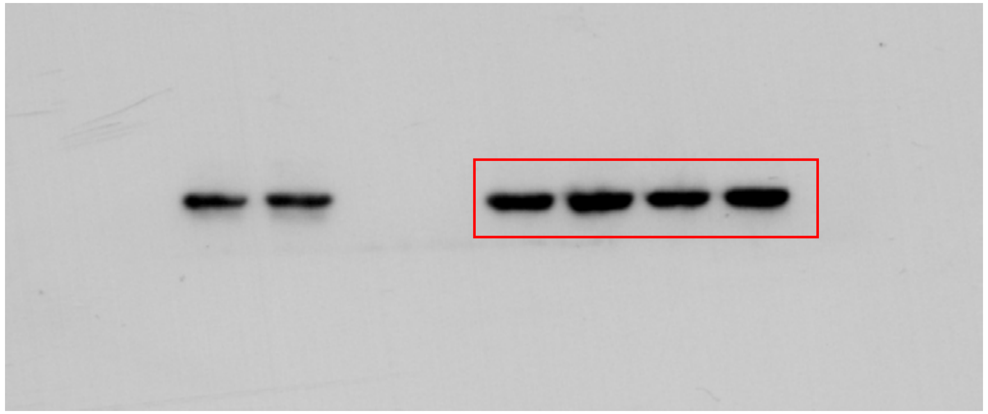

Fig3I Membrane

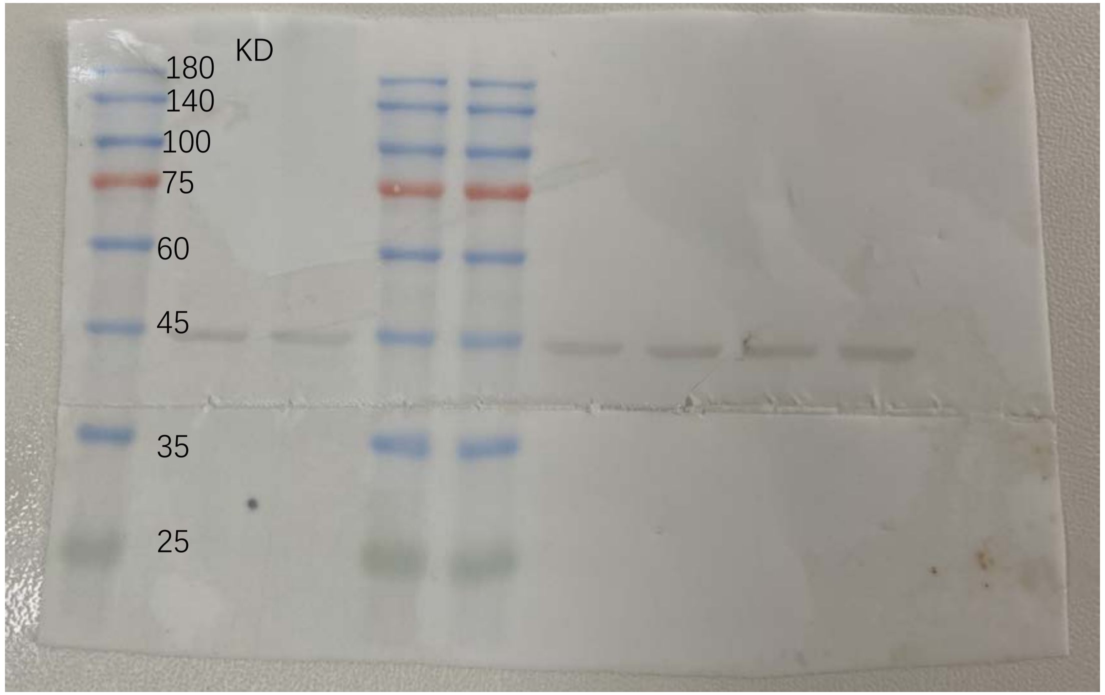

Fig3L β-actin

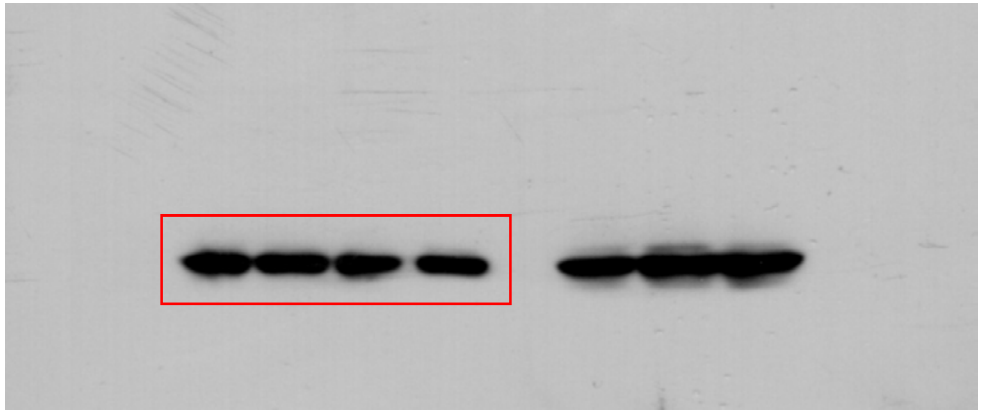

Fig3L GFP

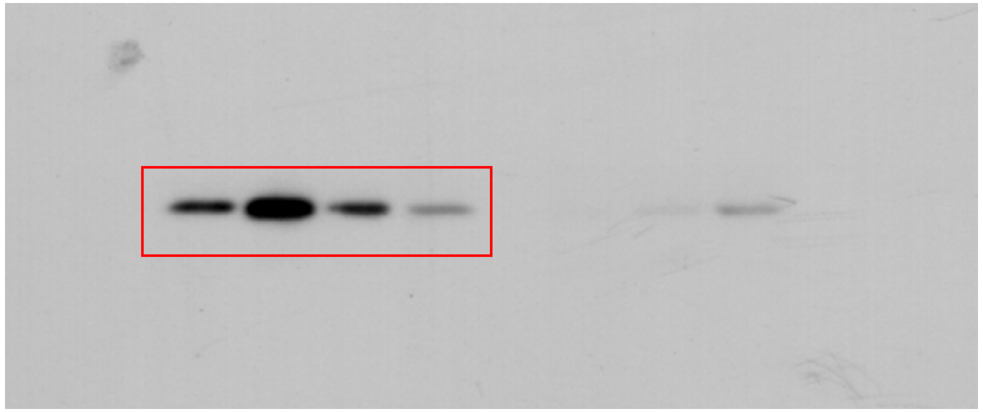

Fig3L Membrane

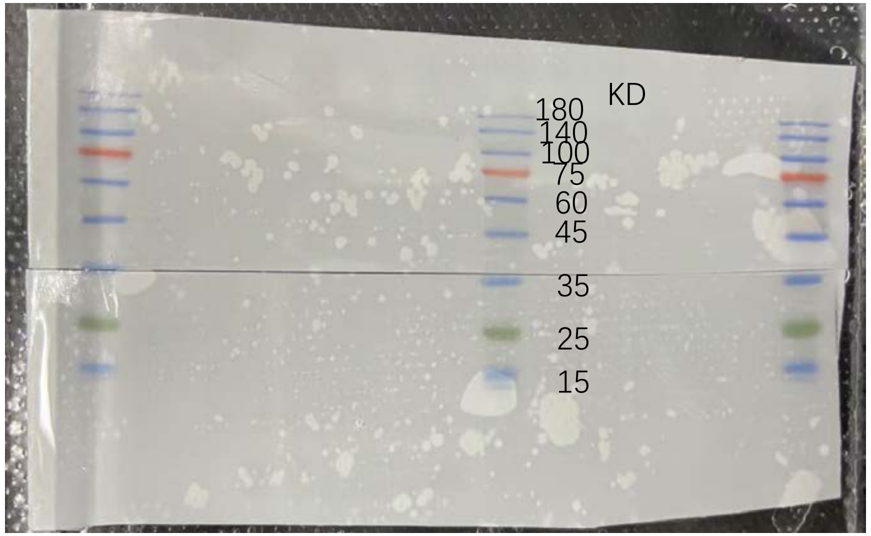

Fig5G β-actin

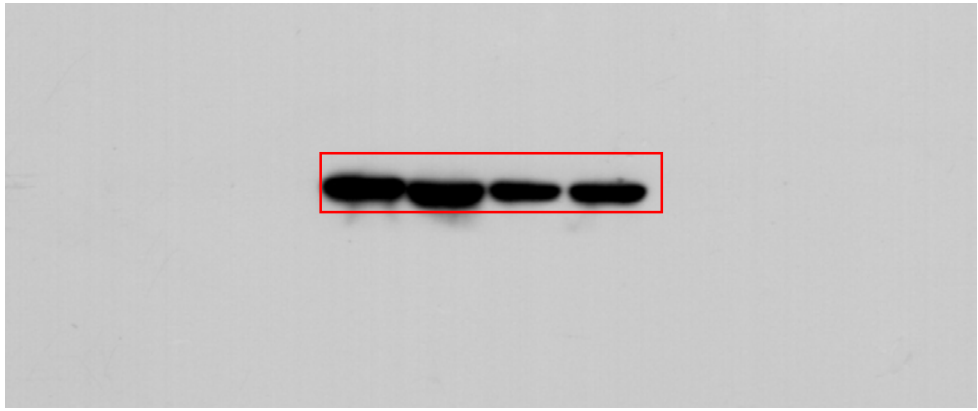

Fig5G GFP

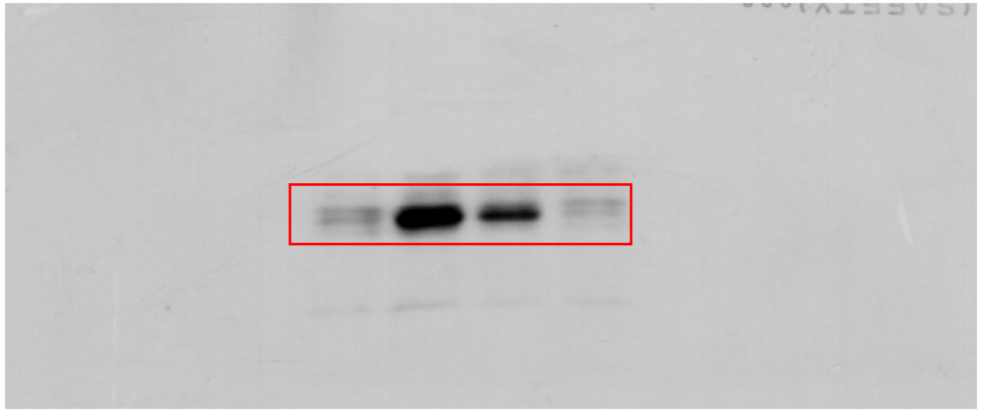

Fig5G Membrane

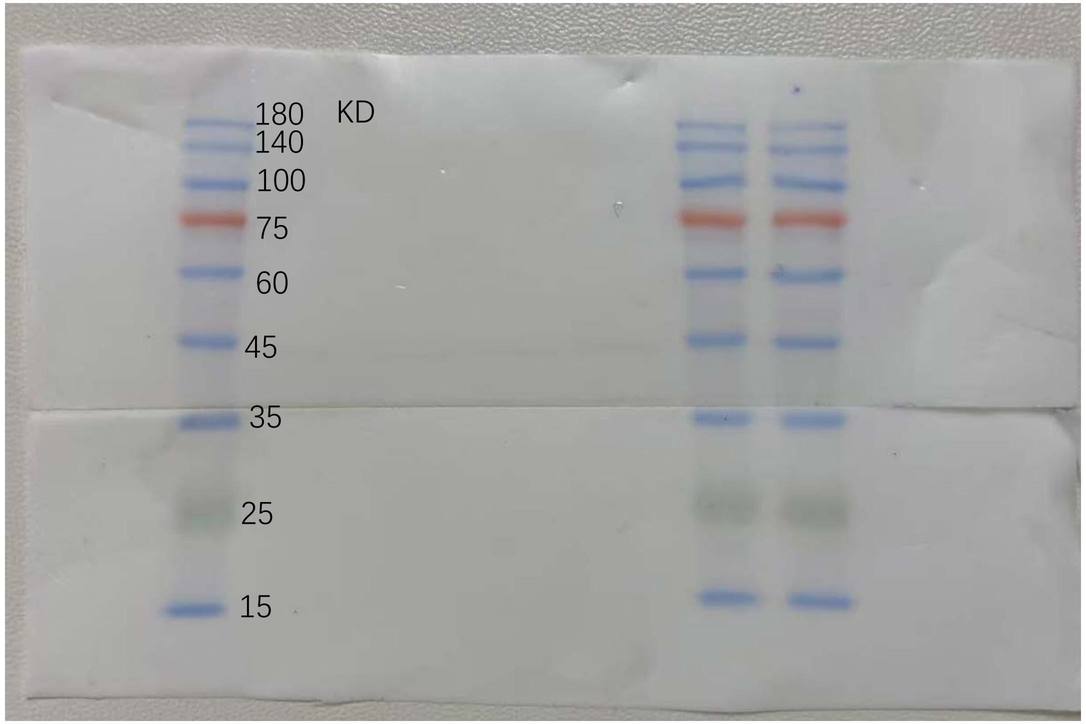

Fig S2E β-actin

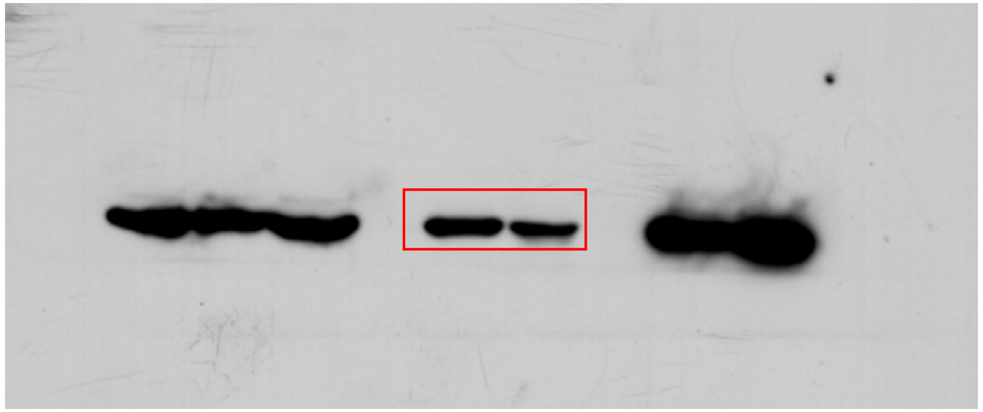

Fig S2E GFP

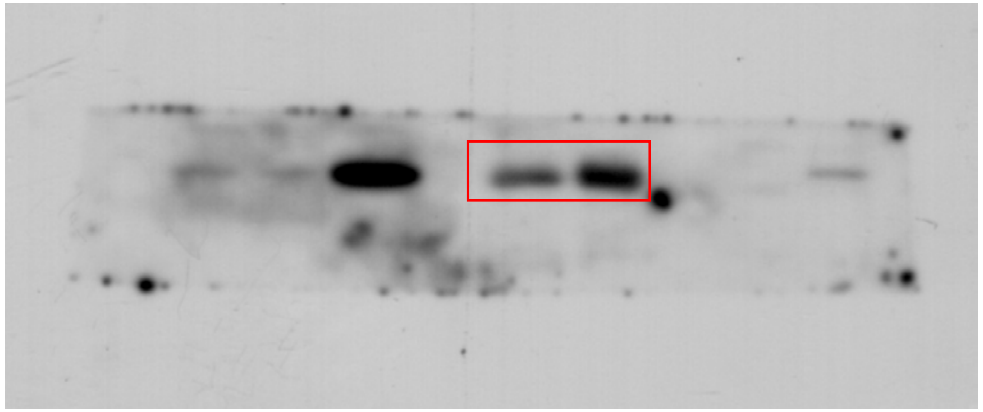

Fig S2E Membrane

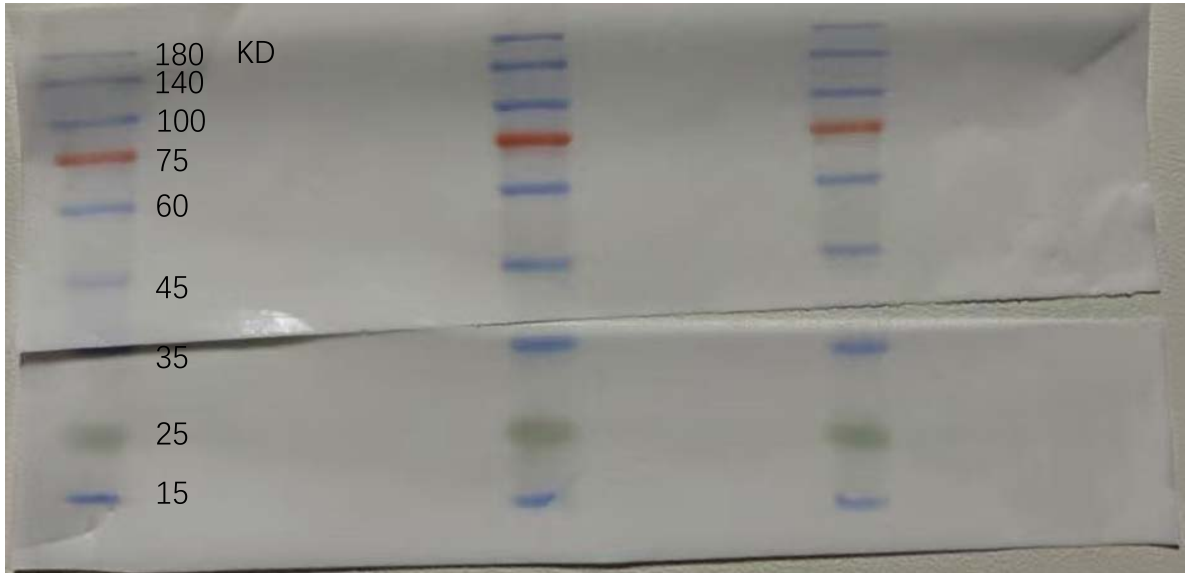

Fig S2F β-actin

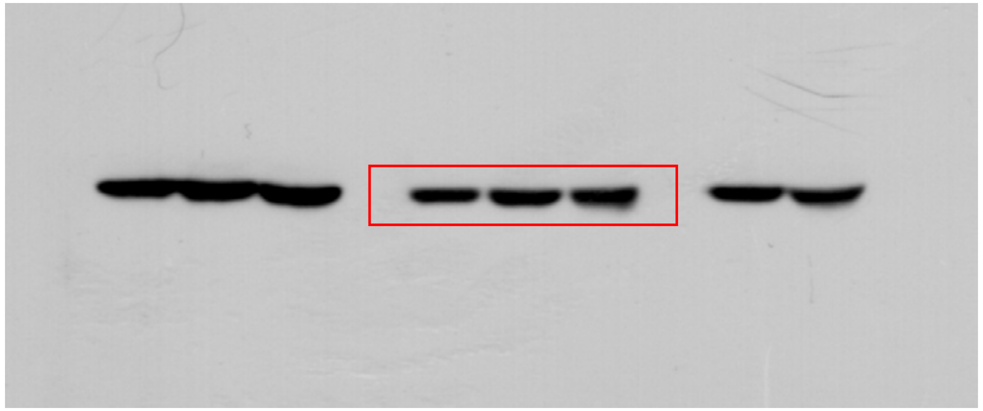

Fig S2F GFP

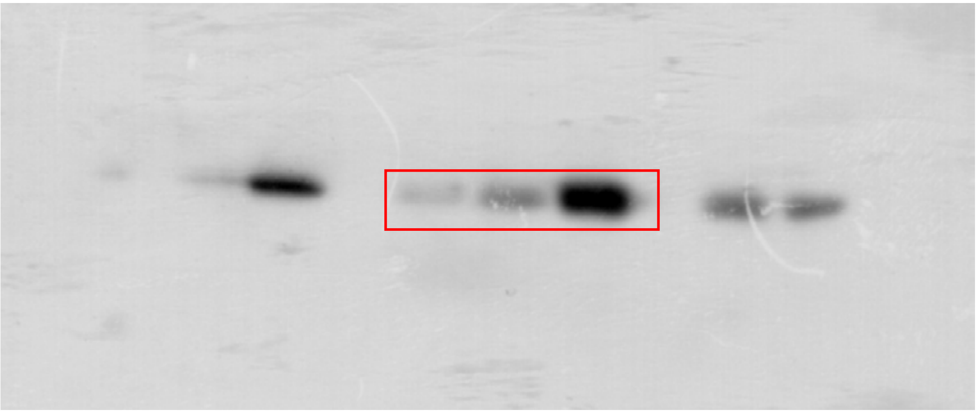

Fig S2F Membrane

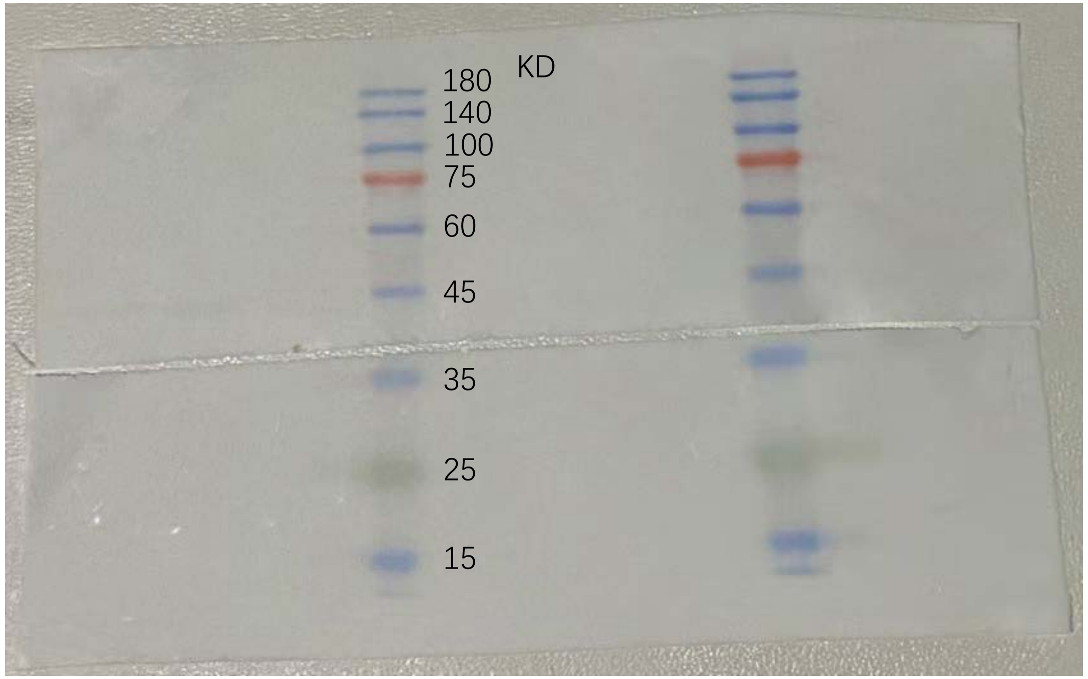

Fig S3H β-actin

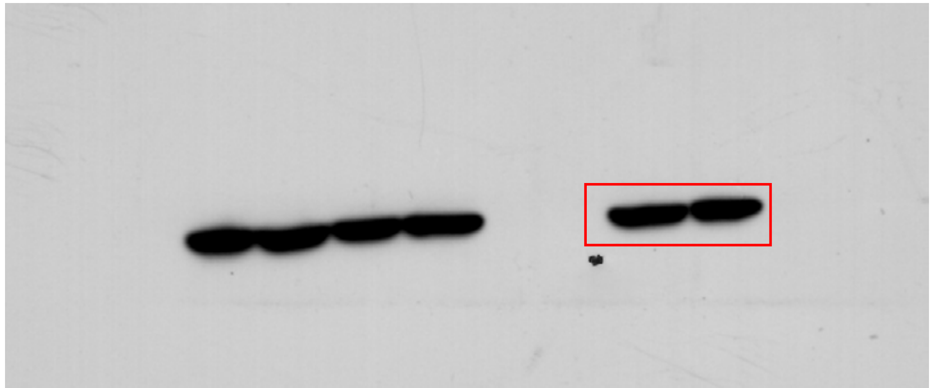

Fig S3H Flag

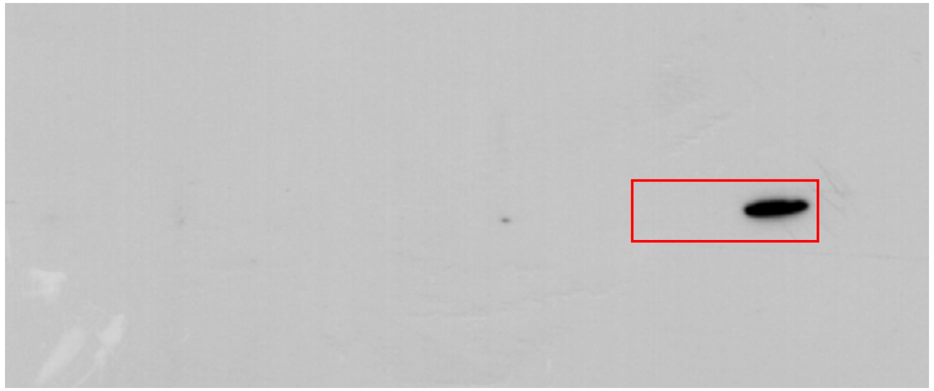

Fig S3H Membrane

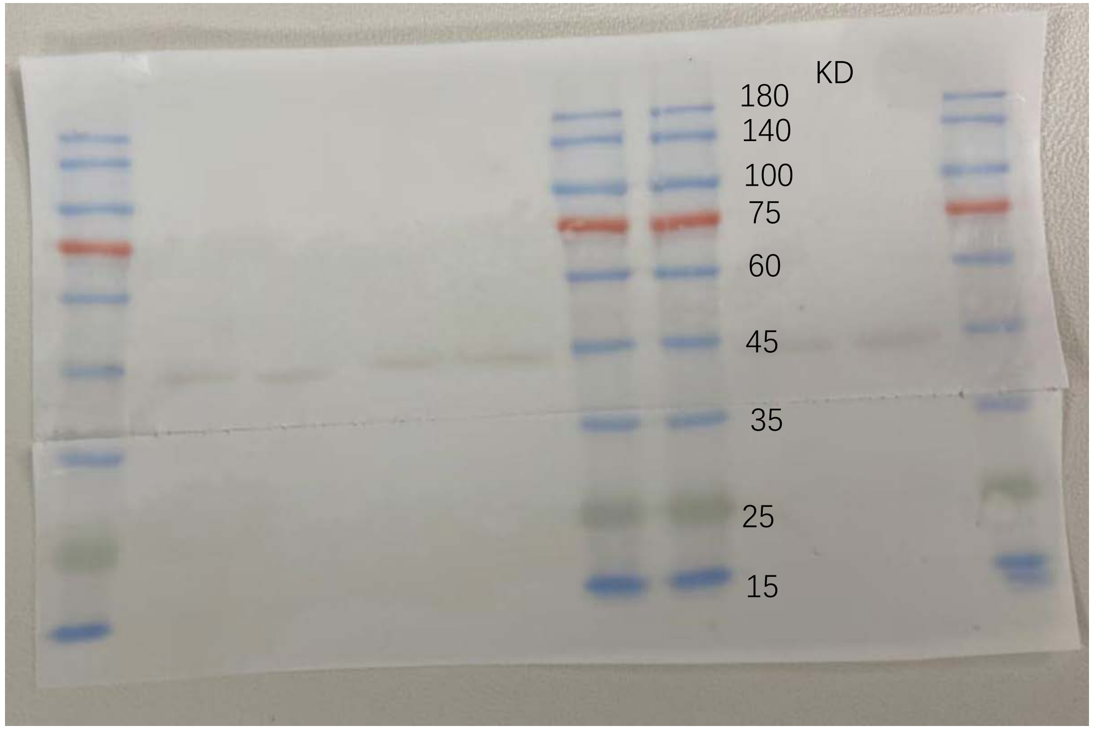

Fig S5D Dlg1

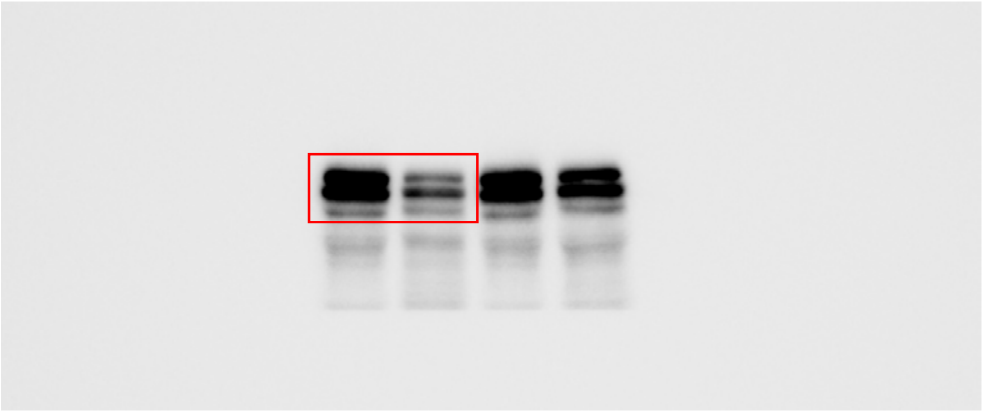

Fig S5D Dlg1-Membrane

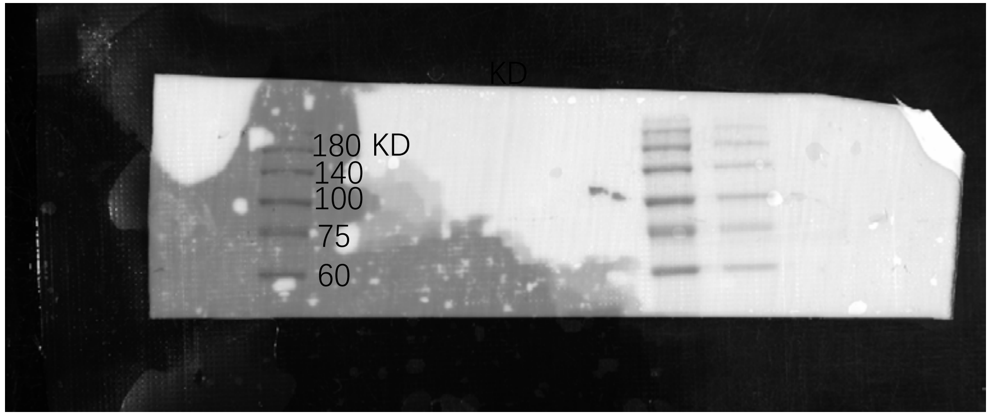

Fig S5D β-actin

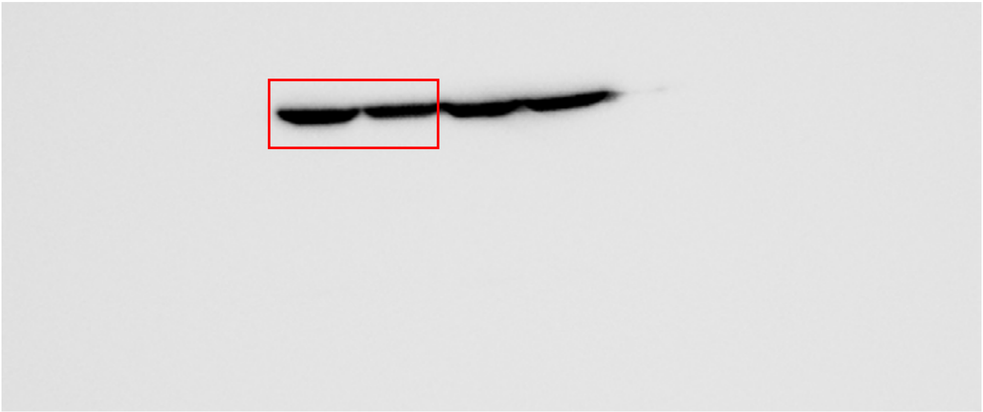

Fig S5D β-actin -Membrane

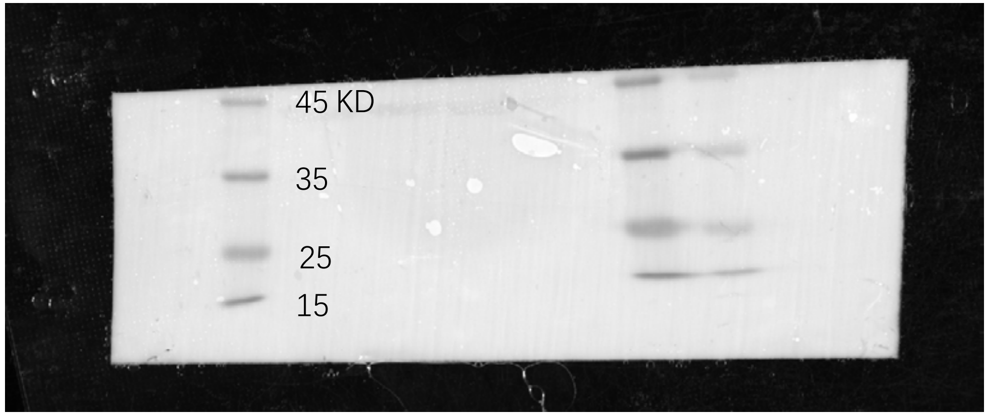

Supplement: S1 File — (ZIP) [file ppat.1012797.s010.zip › uncropped western blot images.pdf]
